# Heat Flux Sensors for Infrared Thermography in Convective Heat Transfer

**DOI:** 10.3390/s141121065

**Published:** 2014-11-07

**Authors:** Giovanni Maria Carlomagno, Luigi de Luca, Gennaro Cardone, Tommaso Astarita

**Affiliations:** Department of Industrial Engineering, University of Naples Federico II, Piazzale Tecchio 80, Napoli 80125, Italy; E-Mails: deluca@unina.it (L.L.); gcardone@unina.it (G.C.); astarita@unina.it (T.A.)

**Keywords:** infrared thermography, heat flux sensors, convective heat transfer

## Abstract

This paper reviews the most dependable heat flux sensors, which can be used with InfraRed (IR) thermography to measure convective heat transfer coefficient distributions, and some of their applications performed by the authors' research group at the University of Naples Federico II. After recalling the basic principles that make IR thermography work, the various heat flux sensors to be used with it are presented and discussed, describing their capability to investigate complex thermo-fluid-dynamic flows. Several applications to streams, which range from natural convection to hypersonic flows, are also described.

## 1. Introduction

The main purpose of this paper is to review on how to take advantage of InfraRed Thermography (IRT) for measuring wall convective heat fluxes, *i.e.*, heat transfer between a body surface and a fluid flowing over it, and to describe some of the IRT applications performed by the authors' research group at the University of Naples Federico II on complex fluid flows.

Measuring heat fluxes is one of the challenging tasks of thermo-fluid-dynamics and requires both a proper *thermal sensor* (which is herein called *heat flux sensor*), with its related thermo-physical model, and, being temperature always involved in heat transfer processes, one or more *temperature transducers*.

In more conventional techniques where temperature is measured with standard transducers (e.g., thermometers, thermocouples, resistance temperature detectors (RTDs), pyrometers, *etc*.), each transducer yields either the temperature at a single point, or its average over a discrete space. Hence, in terms of spatial resolution, the heat flux sensor itself has to be considered as *zero-dimensional*. This constraint makes measurements essentially meaningless whenever the temperature, and/or the heat flux fields exhibit high spatial variations.

Instead, the *infrared* (IR) *camera*, also called *infrared scanner*, constitutes a truly *two-dimensional* temperature transducer since it allows accurate measurements of surface temperature maps even in the presence of relatively high spatial gradients [1]. Accordingly, also the heat flux sensor becomes two-dimensional, as long as the later shown corrections are performed.

Infrared thermography is a methodology which allows for remote detection of thermal energy that is radiated from objects in one of the electromagnetic spectrum infrared bands, conversion of such energy into a video signal, and a two-dimensional representation of the object surface temperature distribution (map). The method can be exploited in many application fields and for many different purposes, as long as surface temperature variations are involved. For example, IRT may be used in various different types of diagnosis (in medicine, architecture, maintenance), or in material characterization and procedures assessment, which can help improve design and manufacturing of products, as well as in non-destructive testing. As the technology evolves, infrared systems offer new prospects for new applications. Almost any process which is temperature-dependent may benefit from the use of an infrared device.

### Infrared Thermography and Thermo-Fluid-Dynamics

As far as convective heat transfer measurements in thermo-fluid-dynamics are concerned, when compared to standard methods, the use of an infrared camera as a temperature transducer for heat flux sensors appears advantageous from several points of view [1].

Since the IR camera is two-dimensional, with current systems having up to about 1 M pixels per frame, besides producing a whole temperature map, IRT allows an easier evaluation of errors due to radiation and tangential conduction in the sensor. The camera does not disturb the phenomena being measured (*non-intrusive*), and so it does not alter conduction through the test article because of embedded sensors and wiring, it has *high sensitivity* (down to 10 mK) and *low response time* (down to 20 μs). As such, IR thermography can be effectively exploited to measure convective heat fluxes with either steady, or transient, techniques.

The major application of infrared thermography in thermo-fluid-dynamics is the measurement of the *convective heat flux q_c_* (energy which flows in the heat mode per unit surface and per unit time, W/m^2^), and/or of the *convective heat transfer coefficient h* (heat flux per unit temperature difference, W/m^2^K), between a solid surface and a fluid flowing over it.

It has to be explicitly pointed out that the heat flux, as such, is a vectorial quantity. However initially, only its convective component normal to exchanging surface *q_c_* is going to be considered.

The link between the quantities *q_c_* and *h* is the well-known *Newton*'*s law* [2]:

(1) $$q_{c}=h\left(T_{w}-T_{r}\right)$$

which is herein written in a generalized form and where *T_w_* is the *surface* (wall) *temperature* and *T_r_* is a *reference temperature* that depends on the fluid flow actual conditions.

For example, for simple hyposonic (*i.e.*, small Mach number *M*) external flows, the reference temperature *T_r_* is the static temperature of the undisturbed fluid. Instead, for high Mach number flows, the correct choice for *T_r_* is the so-called *adiabatic wall temperature* [2,3]. It has to be pointed out that the adiabatic wall temperature does not depend only on the Mach number and should be also used in other cases, e.g., for the mixing of two hyposonic streams at different temperatures, such as a warm jet issuing in cold air and impinging on a plate [4]. On the other hand, for hyposonic internal flows, the correct choice for the reference temperature *T_r_* is the local bulk (cup) temperature in the duct cross section.

It is the present authors' opinion that, regardless of the particular flow conditions, *T_r_* should be always referred to with the generalized concept of local adiabatic wall temperature since, as Equation (1) shows, this is the wall temperature *T_w_* that leads to *q_c_* = 0.

Within its thermo-fluid-dynamic applications, sometimes, infrared thermography is used also from a more qualitative point of view. This occurs when it is important to characterize the flow field behavior (e.g., transition to turbulence, flow separation and reattachment as well as detection of instability phenomena) but this is of limited interest in the present quantitative frame of reference.

Being *h* assumed always positive, consequently in Equation (1) the heat flux is considered to be positive if the energy goes from the solid surface to the fluid, *i.e.*, when the fluid is being heated by the wall along which it flows.

Heat transfer theoretical and experimental data is usually reported in terms of dimensionless quantities; in particular, both the *Nusselt number Nu* and the *Stanton number St* are widely used [5]. These two numbers are conventionally defined respectively as:

(2) $$Nu=\frac{hd}{k_{f}}$$

(3) $$St=\frac{h}{\rho_{f}c_{p}V}$$

where: *d* is a length (m) which, e.g., can be the hydraulic, or equivalent, diameter for internal flows or, in general, a flow characteristic length (such as the gap of an annulus, the distance from a leading edge, the boundary layer thickness, *etc*.); *k_f_* (W/m·K), *c_p_* (J/kg·K) and *ρ_f_* (kg/m^3^) are respectively the fluid thermal conductivity coefficient, specific heat at constant pressure and mass density, all evaluated at film temperature [5]; *V* (m/s) is a reference velocity, which can be the free stream velocity in external flows, the bulk velocity in a pipe flow and so on.

The Nusselt number is generally employed for internal flows while the Stanton number mostly in the case of external flows.

To measure either *q_c_*, or *h*, a thermal sensor, commonly called *heat flux sensor*, is necessary and this justifies the matter treated in this paper for its IRT use.

As it happens when using standard transducers, also for infrared thermography applications, the heat flux sensor generally consists of a slab body with a well-known thermal behavior, whose surface temperature has to be measured by the IR camera. By properly applying to this body a suitable thermo-physical model and the energy conservation equation, it is generally possible to find a relationship between the measured temperature and the convective heat flux, and/or the heat transfer coefficient, between the sensor and the moving fluid.

As far as sensor semantics is concerned, the slab surface the flow is going over, *i.e.*, interacting with the fluid, is herein called *front surface* (its temperature being always indicated with the symbol *T_w_*), while the opposite one *back surface* (which may have a temperature *T_1_* different from the front surface).

When the thermo-physical properties (e.g., thermal conductivity coefficient, specific heat, mass density) of the slab can be considered as independent of its thermodynamic state, the sensor is considered to be *ideal*. Frequently, these properties vary only slightly with temperature so that it is often possible to assume the heat flux sensor as ideal. This hypothesis, together with a constant (in time) reference temperature, is mostly adopted from now on.

## 2. Basics of Infrared Thermography

Infrared thermography is based on radiation heat transfer which is an energy transport mechanism that occurs under the form of electromagnetic waves. By way of this heat transfer mode, energy can travel also in vacuum and may partially be absorbed and reflected by a body, or even pass through it [5]. If the intensity of radiation is put equal to unity, and by denoting with *α_r_* the fraction being absorbed by the body, with *ρ_r_* the fraction being reflected by it and with *τ_r_* the fraction being transmitted (which passes through the body), energy conservation requires:

(4) $$\alpha_{r}+\rho_{r}+\tau_{r}=1$$

where: *α_r_*, *ρ_r_* and *τ_r_* are respectively called *absorptivity*, *reflectivity* and *transmissivity coefficients* of the body under consideration. These dimensionless coefficients may depend on both radiation wavelength (*spectral*) and wave propagation direction (*directional*).

Radiation is emitted by all bodies at an absolute temperature *T* > 0 and, for *opaque* (non-transparent) bodies (*τ_r_* = 0), it originates only from their surface.

The body which emits the greatest amount of energy at a given temperature is called *black body*.

The law [5] setting the energy flux (energy rate per unit area) per wavelength (called *spectral hemispherical emissive power*) emitted by a black body *I_b_*(*λ*) [W/m^3^] is the *Planck*'*s law of radiation*:

(5) $$I_{b}\left(\lambda \right)=\frac{C_{1}}{\lambda^{5}\left(e^{c_{2}/\lambda T}-1\right)}$$

where *λ* is the considered radiation wavelength (m), *T* the absolute black body temperature (K) and *C_1_* and *C_2_ the first and the second universal radiation constants*, equal, respectively, to 3.7418 × 10^−16^ Wm^2^ and 1.4388 × 10^−2^ Km . Equation (5) shows that *I_b_* goes to zero for both *λ* → 0 and *λ* → ∞.

Generally, the electromagnetic spectrum is roughly divided into a number of wavelength *bands*. The infrared spectral band, of interest within the present framework, is generally sub-divided into four lesser bands with subjectively chosen boundaries: *near infrared* (0.75 ÷ 3 μm), *middle infrared* (3 ÷ 6 μm), *long* (or *far*) *infrared* (6 ÷ 15 μm) and *extreme infrared* (15 ÷ 1000 μm). Most of currently used IR cameras are sensitive in the middle (MWIR, 3 ÷ 5 μm) and the long (LWIR, 8 ÷ 12 μm) spectral bands.

By deriving and integrating Equation (5) with respect to *λ*, the following two laws respectively originate:

*Wien displacement law*: The wavelength λ* at which the black body emits its maximum spectral emissive power is a function of the absolute black body temperature *T* according to:
  (6) λ∗T=2897.8μmK
  *i.e.*, the maximum value of *I_b_* moves toward shorter wavelengths as black body temperature increases.
*Stefan-Boltzmann law*: The *total* (over all wavelengths) *hemispherical emissive power E_b_* (W/m^2^) also depends on the absolute black body temperature alone:
  (7) $$E_{b}=\sigma T^{4}$$
  where *σ* is the *Stefan-Boltzmann constant*, which is equal to 5.6704 × 10^−8^ W/m^2^K^4^.

Since IR camera detectors capture only a relatively narrow band of the whole electromagnetic spectrum, Planck's law (5), rather than Stefan-Boltzmann law (7), should be applied to the scanner.

Real objects almost never comply with the above described laws even if they may approach blackbody behavior in certain spectral bands and conditions. A real object generally emits only a fraction *I*(*λ*) of the radiation emitted by the black body *I_b_*(*λ*), at the same temperature and wavelength.

By introducing the *spectral emissivity coefficient*, defined as:

(8) $$ɛ\left(\lambda \right)=I\left(\lambda \right)/I_{b}\left(\lambda \right)$$

Equation (5) can be rewritten for real bodies by simply multiplying its second term by *ε*(*λ*):

(9) $$I\left(\lambda \right)=ɛ\left(\lambda \right)\frac{C_{1}}{\lambda^{5}\left(e^{C_{2}/\lambda T}-1\right)}$$

*Kirchhoff law* states that the spectral emissivity coefficient *ε*(*λ*) is equal to the *spectral absorptivity coefficient α_r_*(*λ*), which is the absorbed fraction of the incident radiation of wavelength *λ*. So, for opaque bodies, such as those mainly used in infrared thermography, Equation (4) becomes:

(10) $$ɛ\left(\lambda \right)+\rho_{r}\left(\lambda \right)=1$$

Therefore, materials with low emissivity *ε* (such as polished metallic materials) not only emit less energy but also reflect a large amount of the radiation, coming from the ambient and impinging on them. Whenever possible, they should not be employed in IR thermography or, should they be used, they have to be sandblasted or, if transient heat transfer is not involved, covered with a thin layer of thermally black paint (such as white dull enamel). Bodies with *ε* independent of *λ* are called *grey*.

Besides, real objects almost never emit in an isotropic (independent of direction, *diffuse*) way, the emissivity coefficient *λ* being dependent also on the angle *θ* between the direction of emission and the normal to the emitting surface (*viewing angle*) [1]. Non-metallic materials, which are mostly used in IRT, emit more nigh to the normal direction.

Within the present context, measurement of convective heat fluxes can be performed with infrared thermography by means of a thermal sensor, in which appropriate surface temperatures have to be measured. As it will be shown in the following section, by correctly choosing a suitable heat flux sensor, IR thermography can be successfully exploited to resolve convective heat transfer distributions over a body surface with either steady, or transient, techniques.

## 3. Heat Flux Sensors

In the following, to define the various heat flux sensors, the concept of *thermal thickness* (thin thermal thickness involves *T_w_* practically equal to *T_1_*, while, for the thick one, *T_w_ ≠ T_1_*), which is more accurately explained in Section 4.2, is used.

With infrared thermography, five different types of heat flux sensors (all under the form of slabs), three steady state and two unsteady, can be essentially employed [1,6,7]. They are:

- *Heated thin foil sensor*. The slab usually consists of a thermally thin metallic sheet (foil), or a printed circuit board, steadily and uniformly (in space) heated by Joule effect [1,6]. Strictly speaking, the foil may be heated also in a different way (e.g., by a radiation heat flux impinging on the foil) but then, the heat flux distribution should be precisely known. The convective heat transfer coefficient can be computed by measuring the heat input, as well as the foil surface temperature with the IR scanner, and by performing a complete energy balance. Due to the foil thermal thinness, the temperature can be measured on either one of the slab surfaces but it is possible to apply this steady-state sensor also to not thermally thin foils (see Section 4.2).
- *Gradient sensor*. In this steady-state sensor, the slab is thermally thick and the temperature difference across the slab thickness is measured. Then, by knowing this thickness and the thermal conductivity coefficient of the slab, the heat flux across it can be computed [6]. In conventional methods, the temperature difference is usually measured by a thermopile of very-thin-ribbon thermocouples or by two thin-film resistance thermometers. To make easier the use of this sensor with infrared thermography, its back surface could be maintained at a given constant temperature (e.g., in contact with a heat sink) so only the front surface (which must be viewed by the scanner) temperature has to be measured.
- *Laplacian sensor*. An unusual steady-state method for measuring convective heat transfer coefficients has been recently described and tested by Carlomagno *et al.* [7]. The proposed technique can be applied to thermally thin sensors, such as slabs made of relatively high thermal conductivity material. Unlike the heated thin foil method demanding a given uniform heating of the slab, this steady sensor is externally (out of the measuring zone) heated, and the heat input is not even to be known. In the energy balance, the tangential conduction (parallel to slab surface) and the convective heat fluxes result to be the predominant contributions. Since currently available IRT allows measuring the two-dimensional temperature distribution with relatively high spatial resolution, this occurrence makes it possible to evaluate the tangential conduction by numerical computation of the temperature distribution Laplacian value. Spatial filtering with a Gaussian window and computation of the numerical derivatives with a relatively large step are needed to deal with the unavoidable noise presence in the acquired data.
- *Thin film sensor*. A thermally thick slab is used as a sensor and the convective heat transfer coefficient is inferred from the theory of unsteady heat conduction in a semi-infinite solid, having a thermal input at its surface. The name of the sensor classically derives from the thin resistance thermometer (typically a platinum very thin film), which is bonded to the low conductivity slab surface. Clearly, the thin film must have negligible heat capacity and thermal resistance as compared to the slab layer affected by the exchanged heat flux. When this unsteady sensor is used in combination with an IR camera, the thin resistance thermometer does not exist but the slab surface in contact with the exchanging fluid must be necessarily viewed by the IR scanner.
- *Thin skin*, or *wall calorimeter sensor*. The sensor is made of a thermally thin slab (skin) and is used as a perfect calorimeter. Being the slab thermally thin, the temperature can be assumed to be constant across its thickness and the convective heat flux is evaluated from the time rate of the slab temperature change. The use of this sensor with IR thermography is straightforward and either one of the slab surfaces can be viewed by the IR scanner. Furthermore, as for the heated thin foil, it is relatively easy to make the slab quite thin because it is not required to include, in this unsteady sensor, also a temperature transducer, such as a thermocouple or else.

To summarize, the above described five types of one-dimensional sensors involve the measurement of the quantities reported in Table 1, where those in the last two lines are functions of time *t* and those in the third column refer to thermally thin sensors.

A widely-used classical heat flux sensor is the Gardon gauge (also Schmidt-Boelter) which is typically a zero-dimensional sensor, so of no interest herein. Quite recently, a different type of heat flux sensor, based on a numerical solution of Fourier's law (typically described by an inverse heat transfer model) and surface temperature measurements, has been developed (e.g., Roger [8]). The advantage of using such an approach is that it is possible to take into account the temperature dependence of the material thermo-physical properties and/or to have slabs with high curvatures. However, for the sake of simplicity, this heat flux sensor will not be herein described.

With conventional transducers, it is possible to measure the wall temperature only in a relatively small number of discrete points. Thus, to perform reliable measurements, the hypothesis that, at each point, the heat flux across the slab has to be considered as one-dimensional is normally needed. This requirement obviously implies that the heat flux vector to be measured must be normal to the sensing element surface, *i.e.*, that the components of the temperature gradient, which are parallel to the slab surface (tangential), can be neglected and so the sensor is practically zero-dimensional.

The one-dimensional hypothesis can be dropped when the surface temperature is measured with an infrared scanner because of the high data number and spatial resolution of the measurement. This subject is later addressed and discussed with more details, including the needed corrections.

Very often, the heat flux sensor is flat; however in practice, the slab surfaces can also be curved, but their curvatures can be ignored as long as the thickness of the layer affected by the input heat flux is relatively small as compared with the local radius of curvature of the sensor front surface.

Even if the heat flux, or the convective heat transfer coefficient, may be considered as constant over time, both the thin film and the wall calorimeter intrinsically involve an unsteady measurement procedure of temperature. They definitely operate with a so-called *passive* heating which is due to some already existing temperature difference between the sensor front surface and the flowing fluid. Instead, the heated thin foil, the gradient and the Laplacian sensors are, generally, connected to a steady state procedure that requires some *active* heating.

As a general comment, it has to be pointed out that, while examining gas flows, with the thermally thin heated thin foil, Laplacian or thin skin sensors, one may generally measure the temperature map on either the front, or the back, surface of the sensor (in case, performing the required corrections).

Instead, if liquids are involved, the back surface must be generally viewed by the scanner because liquids are not usually transparent to infrared radiation. This means that thin film and gradient sensors cannot be used in liquid flows because front surface temperature cannot be detected by the camera.

In the following, first the steady sensors and then the two unsteady ones are discussed, all for steady convective heat flux and/or heat transfer coefficient (Sections 4–8); afterwards, the application of some of them to a few thermo-fluid-dynamic problems are presented and discussed in Section 9.

## 4. Heated Thin Foil Sensor

### 4.1. Basics

With IR thermography, the simplest steady state sensor that allows us to measure convective heat transfer coefficients, is the heated thin foil sensor which mostly constitutes also the slab front surface.

**Figure 1.**
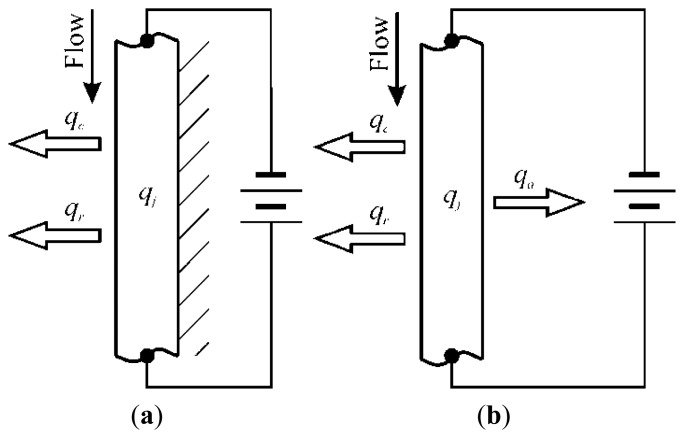
Sketch of the heated thin foil sensor: (**a**) adiabatic back surface; (**b**) diabatic back surface.

**Figure 2.**
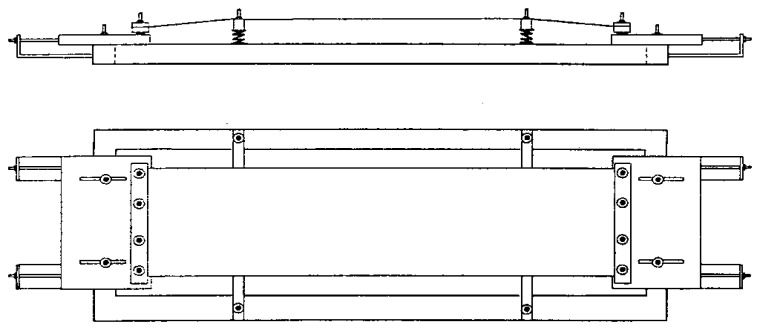
Sketch of a foil heater for natural convection studies. From Cardone and Carlomagno [9].

Referring to the sketch of Figure 1a, where the thickness is represented much larger, in its classical and simplest accomplishment, the sensor consists of a thin metallic foil (frequently a stainless steel, or constantan, foil, typically tens of microns thick [1,9], e.g., see Figure 2 where thickness is represented to scale), steadily and uniformly (in space) heated by Joule effect. The foil is often thermally insulated at its back surface (right vertical surface in Figure 1a and inner surface of Figure 2) while its front surface is exposed to the fluid stream.

The constant electric potential difference to the foil can be practically achieved by using a stabilized DC power supply and two couples of bus bars, usually made out of copper, which, as shown in Figure 2, are clamped at two opposite foil edges. The very large equivalent cross section (weighted with small electrical resistivity) of the bus bars, with respect to that of the heated foil, should ensure that the voltage drop along them is very small. In Figure 2, it can be seen that foil wrapping and thermal expansion are taken care of by the lateral stretching screws and the existing springs pushing two rods.

**Figure 3.**
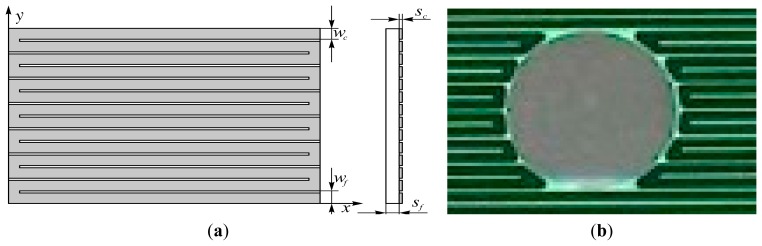
(**a**) Heated thin foil sensor realized with a printed circuit board; (**b**) Close-up of a printed circuit board with a 24 mm hole to study a jet in cross flow (*w_f_* = 2 mm, *w_c_* = 1.8 mm) [12].

Since the geometries achievable with a thin metallic foil (which has to be uniformly heated) are quite limited (practically, only the rectangular ones), an easily attainable extension of this sensor is to use a printed circuit board as a heating element. Often in this case, the closely spaced copper tracks are 5 to 35 μm thick and arranged in a Greek fret mode over a fiberglass support (see Figure 3a) [10,11].

In both implementations, it is extremely easy to obtain also cylindrical geometries (e.g., see Section 9.5).

A geometry conical and/or with the presence of holes (e.g., see Figure 3b) can be attained in the case of printed circuit boards, while more complicated shapes may be achieved by patching together different *clothes*, as long as the sensor surface has not a high double curvature.

In principle, the foil could be heated by any mean (e.g., also by radiation) and even in a non-uniform way, but in such a case, the input heat flux should be precisely known in every point of the sensor. In many instances, this measurement could be accomplished with the evaluation of the heating distribution by using the foil also as a thin skin sensor (see Section 8).

For the sake of simplicity in the following, it is always supposed that the heating is uniform in space and constant in time.

Apart from the later discussed influence of the electrical resistivity temperature coefficient, from the heat transfer point of view, the Joule heated thin foil experimentally provides an almost constant convective heat flux boundary condition.

By assuming that the back surface of the sensor is adiabatic as in Figure 1a, it is easy to perform a simple steady state one-dimensional energy balance per unit area of the sensor and per unit time:

(11) $$q_{j}=q_{c}+q_{r}$$

where: *q_j_* is the imposed input Joule heat flux (see Equation (17)), *q_c_* is the convective heat flux to the flowing fluid and *q_r_* is the radiative heat flux to the ambient environment on the fluid side. This latter contribution unavoidably exists (because the sensor front surface must be seen) and, from a practical point of view, it has to be considered as a heat loss.

The presence of *q_r_* applies to all sensor types where temperature is measured by infrared thermography over one of the sensor surfaces.

By assuming the fluid ambient environment as a black body at a constant temperature *T_a_* and that the sensor surface is a grey one (assumptions which can be made in most of the cases for the involved wavelengths), the radiative heat flux can be computed by using Equations (7) and (8):

(12) $$q_{r}={ɛ}_{t}\sigma \left(T_{w}^{4}-T_{a}^{4}\right)$$

where *σ* is again the Stefan-Boltzmann constant and *ɛ_t_* is the front surface total hemispherical emissivity coefficient. Of course, the radiative heat flux has to be computed point by point of the sensor surface by means of the measured local *T_w_*.

When more conventional techniques are used to measure the wall temperature, it is possible to have a very low wall emissivity coefficient in the involved wavebands (e.g., by gold plating the exchanging surface) so as to neglect the radiative heat flux to ambient environment. Obviously, this is not the case when measuring temperatures of any surface by means of infrared thermography (the surface emissivity has to be high) and, besides, to view this surface, the adjacent fluid has to be (at least, partially) transparent in the used IR detector band.

From the knowledge of *q_j_*, *ɛ_t_*, *T_a_* and *T_r_* and by measuring *T_w_*, through the two previous Equations (11) and (12) and making use of the Newton's law (1), it is possible to directly evaluate the convective heat transfer coefficient [1]:

(13) $$h=\frac{q_{j}-{ɛ}_{t}\sigma \left(T_{w}^{4}-T_{a}^{4}\right)}{T_{w}-T_{r}}$$

By looking at Equation (13), it can be affirmed that, if the radiation contribution can be neglected in the energy balance, the adiabatic wall temperature *T_r_* may be measured for *q_j_* = 0, *i.e.*, by switching off the Joule power feeding the foil.

As later shown in Section 4.2 for this sensor, under the assumption that the total *Biot number*, *Bi* = *h_t_s*/*k_f_* (where *s* and *k_f_* are respectively the thickness and the thermal conductivity coefficient of the sensor and *h_t_* includes both convection and radiation) is quite small as compared to unity, temperature can be considered practically constant across the foil thickness.

In this instance, it is also possible to measure the temperature of the back surface of the sensor, which, being thereupon viewed obviously becomes diabatic (see Figure 1b).

This occurrence is very convenient when using IR thermography in liquid flows since most liquids are opaque in the used IR bands or whenever the sensor front surface is not accessible to the IR scanner for any other reason.

When also the back surface is diabatic (see Figure 1b), Equation (13) has to be extended by subtracting from *q_j_* also the heat loss *q_a_* from this surface to the external ambient environment, so obtaining [1]:

(14) $$h=\frac{q_{j}-q_{a}-{ɛ}_{t}\sigma \left(T_{w}^{4}-T_{a}^{4}\right)}{T_{w}-T_{r}}$$

The heat flux towards the external ambient environment via the back surface is usually the sum of radiative and natural convection heat fluxes. Radiative heat flux can again be appraised by means of Equation (12) with the proper external ambient temperature, while convective heat flux to external ambient may be evaluated according to the existing situation by using standard correlations tables [13–15].

However to more carefully evaluate *q_a_*, it is much better to perform some *ad hoc* tests with the same IR scanner by thermally insulating the front surface of the sensor [16]. The main advantage of performing such kind of tests is that they include the radiative contribution towards the external ambient environment as well.

It has to be explicitly pointed out that both the heat losses *q_a_* and *q_r_* are to be considered as correction terms and, in order to obtain accurate data, they should be a small fraction of the total Joule heating; otherwise, an error in their evaluation could produce a significant error in the measured *h*. This may be particularly true when performing natural convection studies with gases, where *q_r_* can be of the same order of magnitude of *q_c_*.

### 4.2. Limits of the Isothermal Assumption

Often, when using the heated thin foil heat flux sensor, it is possible to detect only the back surface temperature of the sensor, *i.e.*, that opposite to the one the fluid is going over. Since in Equation (1) the front surface temperature *T_w_* must be used, it is necessary to examine what are the limits of the sensor isothermal (across its thickness) assumption.

From a practical point of view, two possible foil heating conditions are examined in the following [1]. The first one regards the case where the imposed heat flux derives from bulk Joule heating of the foil. The second one is encountered when this flux occurs because of an external radiative heating or, in any case, the heat release arises at the sensor back surface. For both conditions, it is assumed that the sensor is ideal and that the measured surface is always the back one.

In the first condition, the heat flux *q_j_* is due to the uniformly generated (except than for small variations due to the temperature coefficient of electrical resistivity) energy rate per unit volume *G* (heat generation, W/m^3^) inside the sensor wall because of the electric current passing through it.

In terms of limits of isothermal condition and unless there is a heat input at the back surface, the worst case is when the back surface is adiabatic while the front one (*i.e.*, that exchanging energy with the fluid) is diabatic. For such boundary conditions, the solution of the steady state Fourier equation leads to the following parabolic temperature distribution inside the sensor:

(15) $$T-T_{w}=\frac{G}{k_{s}}\left(sx-\frac{x^{2}}{2}\right)$$

where *k* is the thermal conductivity coefficient of the foil material, *s* the foil thickness and the coordinate *x* starts from the sensor front surface being directed towards the back one.

Therefore, the maximum temperature difference inside the slab is that which occurs between the front surface *T_w_* and the back one *T_1_* and is equal to:

(16) $$T_{1}-T_{w}=\frac{Gs^{2}}{2k_{s}}$$

In the meantime, the heat flux exchanged with the fluid is equal to:

(17) $$q_{j}=Gs=\frac{2k_{s}}{s}\left(T_{1}-T_{w}\right)$$

By neglecting the radiative contribution *q_r_*, the above quantity must be equal to the convective heat flux at the fluid side given by Equation (1) and, by recalling the definition of the Biot number, it is obtained:

(18) $$Bi=\frac{hs}{k_{s}}=2\frac{T_{1}-T_{w}}{T_{w}-T_{r}}$$

Therefore, the Biot number can be regarded as a measure of the relative importance of the temperature difference in the sensor with respect to that between the sensor itself and the fluid.

In a sense, the Biot number measures the above mentioned thermal thickness of the foil (slab). A relatively low Biot number implies an essentially constant temperature across the foil and, therefore, its low thermal thickness. This is true also in the case of no Joule heating as for the thin skin and the Laplacian sensors.

With regard to the second situation, when the Biot number can get to significant values (*i.e.*, the foil is not thermally thin) it is still possible to detect the temperature from the back surface of the sensor but, in this case, an extension of Equation (14) is needed [17].

E.g., this extension is necessary if the sensor is made of a printed circuit board, or the metallic foil is bonded to a support, and the heating element is placed at the sensor back surface. These two configurations are usual dealing with corrosive (or electrically conducting) fluids and, for this reason, the metallic heating element must be protected. It is easy to imagine that a similar situation may also develop while heating a thermally thick foil by radiation with an external lamp or else.

**Figure 4.**
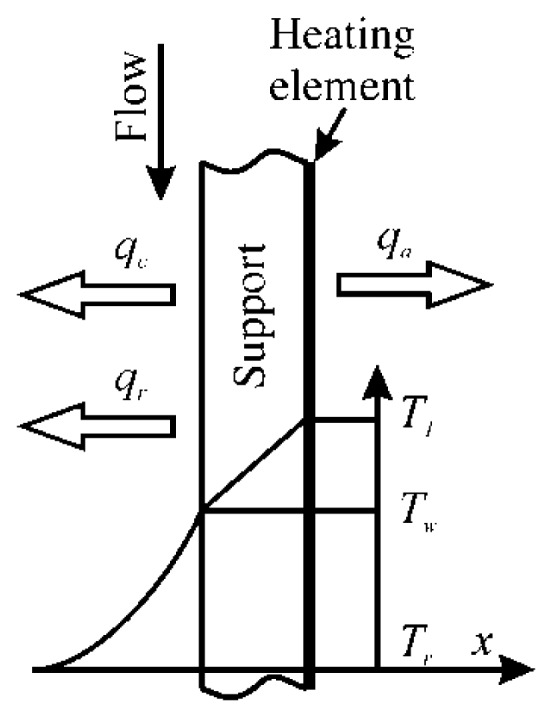
Thin foil heated from the sensor back surface for a relatively high *Bi*. Adapted from [1].

The related geometry is sketched in Figure 4. In this case, it is reasonable to assume that at least the Biot number of the metallic heating component can be considered very small, which is easy to achieve both on account of its thinness and because of its relatively high thermal conductivity coefficient. Of course, in radiation heating the metallic heating element does not exist.

By assuming the sensor ideal, the steady state energy balances applied to both its front and back surfaces become:

(19) $$q_{c}+q_{r}=k_{s}\frac{dT}{dx}=q_{j}-q_{a}$$

where *x* is the spatial coordinate, normal to the sensor surface that is directed away from the fluid, *k_s_* is the support thermal conductivity coefficient, which is assumed to be precisely known, and the derivative is clearly the same on both support sides.

Under the hypothesis of one-dimensional steady heat flux, the derivative of Equation (19) can be easily calculated by noting that the temperature profile within the support thickness *s* may be considered as a linear one:

(20) $$h\left(T_{w}-T_{r}\right)+q_{r}=\frac{k_{s}}{s}\left(T_{1}-T_{w}\right)=q_{j}-q_{a}$$

Since the IR scanner detects the heating element temperature *T_1_* (at the back surface), the previous relation consists of a system of two equations (generally, non-linear) in the two unknown quantities *h* and *T_w_* that can be easily solved by using standard methods.

### 4.3. Tangential Conduction within the Sensor

The assumption of a one-dimensional heat flux sensor is strictly satisfied only if the temperature within the slab constituting the sensor has negligible gradient components along the sensor surface (*i.e.*, in the tangential to surface directions). However, the infrared camera allows researchers to better explore 2D effects, especially in studying complex thermo-fluid-dynamic phenomena, where the temperature of the sensor generally varies over its measured surface. For a heated thin foil sensor made of isotropic material (such as a thin metal sheet), by retaining the assumption that the slab is thermally thin (*i.e.*, isothermal across its thickness) and ideal, it is possible to evaluate the tangential conduction heat flux (*i.e.*, referred to the unit sensor area, W/m^2^) by means of the Fourier's law [1]:

(21) $$q_{k}=-sk_{s}\nabla^{2}T_{w}$$

where: ∇^2^ is the two-dimensional Laplacian operator evaluated in the heat flux sensor plane, *s* and *k_s_* are the thickness and the thermal conductivity coefficient of the foil, respectively.

When using an infrared scanner, a two-dimensional detailed distribution of the surface temperature is directly measured and, in principle, it should be straightforward to evaluate the conductive heat flux by numerically approximating the two-dimensional Laplacian of Equation (21) with the classical 5 points formula. However, the infrared scanner unavoidably embodies in its signal high frequency random noise that is obviously amplified by the numerical derivatives. Therefore, it is indispensable to calculate the Laplacian only after an adequate filtering of the temperature map. One procedure for this signal smoothing can be accomplished by a simple convolution with a Gaussian-filtering window.

In any case, when performing a steady state measurement, to reduce the random noise, it is always very helpful to make an average of several temperature maps acquired in a sequence.

Another kind of smoothing can be accomplished before derivation in some peculiar situations. E.g., when experimental conditions involve temperature variations in only one direction along the sensor surface (such as testing a two-dimensional airfoil in a wind tunnel, or the example of Figure 2), a span wise average can be performed, even for unsteady conditions.

Instead, when the investigated geometry exhibits an axial symmetry (e.g., a jet normally impinging on a flat surface or a disk rotating in still air [1]), it is advantageous to perform an azimuthal average of the temperature map so as to calculate the convective heat transfer coefficient only along the radial coordinate *r* (radial profile). In particular, in this case the Laplacian of Equation (21) reduces to the much simpler formula:

(22) $$\nabla^{2}T_{w}=\frac{\partial^{2}T}{\partial r^{2}}+\frac{1}{r}\frac{\partial T}{\partial r}$$

which, however, has a singularity at *r* = 0 that has to be carefully handled.

Once the tangential conduction heat flux is evaluated with Equation (21), it is easy to extend the approach presented in the previous chapter to the multi-dimensional case. In fact, by including the tangential conduction additional term in the energy balance (in particular, in Equation (14), where the tangential conduction does not exist), the convective heat transfer coefficient can be evaluated by means of [1]:

(23) $$h=\frac{q_{j}-q_{a}-{ɛ}_{t}\sigma \left(T_{w}^{4}-T_{a}^{4}\right)+sk_{s}\nabla^{2}T_{w}}{T_{w}-T_{r}}$$

It will be later shown that Equation (23) allows us to generalize the heated thin foil sensor to a more comprehensive and less complex heating condition, *i.e.*, the Laplacian sensor, by putting *q_j_* = 0.

As previously mentioned, in many practical realizations of the heated thin foil sensor, a spatially quite constant Joule heating can be achieved by using a copper-clad laminate where a printed circuit is carved, *i.e.*, a printed circuit board. The printed circuit board is generally made of a not electrically conductive fiberglass support (typically 0.2 ÷ 0.5 mm thick) to which extremely thin (generally from 5 to 35 μm thick) conductive pure copper tracks are bonded. However, notwithstanding the copper layer thinness, because of the extremely high thermal conductivity coefficient of the copper (*k* ≅ 390 W/mK), the board exhibits an anisotropic thermal conduction behavior (along, or across, the tracks). Therefore, in order to effectively evaluate the tangential conduction term, it is necessary to generalize Equation (21) as extensively reported in [1,16].

### 4.4. Data Analysis and Experimental Procedure

For what already said in Section 4.2, when using the electrically heated thin foil and if the Biot number is not very small, to evaluate *T_w_* to be used in Equation (1) it is necessary to subtract the quantity *Gs*^2^/2*k* from the measured *T*_1_. If it is necessary to consider also the heat losses to ambient environment *q_a_*, computation gets more involved. Luckily enough, when using the electrically heated metallic thin foil, the Biot number turns out to be often very small (generally, less than 1%) so that such a correction is not usually required. Instead, for the second situation described in Section 4.2, a correction according to what suggested there has to be performed.

The other operations to correct acquired data, such as taking into account the radiation terms or the heat flux to ambient environment, are already comprehensively addressed in Section 4.1.

As far as the experimental procedures are concerned, the simple ones described in the following may improve the process of obtaining more reliable data.

As an absolute and often overlooked general comment, it must be pointed out that, as previously affirmed, being the heated thin foil, a steady technique, most of the times it is strongly recommended to first acquire with the IR scanner a relatively large number of temperature distributions (*thermograms*) and then to average them in order to decrease the existing temporal random noise. Of course, in doing so the flow conditions must be kept rigorously constant.

Furthermore, it is suggested that, in hyposonic external flows (which is an often encountered situation), it is much better to previously measure the adiabatic wall temperature *T_r_* of Equation (1), without heating the foil but with on-going normal testing flow conditions, before measuring temperatures while heating the foil. This procedure can compensate the IR scanner limited thermal accuracy (which is worse than its sensitivity), so eliminating the unavoidable bias.

The heat transfer boundary condition that the heated thin foil practically enforces is the constant heat flux one. However, since the convective heat transfer coefficient is not generally uniform over the whole foil, temperature differences may arise (see Equation (1)). They induce local changes of the electrical resistivity of the foil material that, in turn, may produce local variations of the power dissipated by Joule effect. Clearly, this effect is strictly linked to the magnitude of the temperature variations over the foil and to the temperature coefficient of the electrical resistivity but it can be normally neglected by using a proper material (e.g., constantan or manganin alloys).

However also for different types of alloys, it has to be evidenced that, when the temperature differences over the sensor are relatively small, this effect is often negligible. E.g., for a standard nichrome alloy, the temperature coefficient is of the order of half a thousandth per Kelvin and, if the maximum temperature difference over the foil turns out to be not larger than 20 K, the error in the heat flux evaluation is less than 1%.

Instead, for printed circuit boards, it has to be stressed that copper metal has a relatively high temperature coefficient of electrical resistivity (about 0.004 K^−1^), about ten times that of nichrome, *i.e.*, a temperature difference of 20 °C would cause an uncertainty in the heat flux evaluation of about ±4%. Therefore while conducting experiments with printed circuit boards, large temperature differences should be avoided or, should they exist, an adequate correction must be performed on the basis of the measured temperature distribution of the circuit.

A very simple and direct way to accomplish this objective is to multiply, segment by segment of the circuit, the initially estimated Joule average heat flux by the sum of one plus the product of the copper electrical resistivity temperature coefficient times the difference between the locally measured temperature and the mean (over the whole heated board surface) one.

## 5. Gradient Sensor

### 5.1. Basics

As already stated, for this steady state sensor, the temperature difference across the sensor thickness is measured and, by knowing the thickness *s* and the thermal conductivity coefficient *k_s_* of the slab, the heat flux across it can be computed [6,18].

Obviously, the assumption that the slab thermal conductivity coefficient does not depend on temperature makes linear the temperature profile across the slab thickness.

Therefore, the energy balance equation to be used for the gradient sensor is:

(24) $$q_{c}+q_{r}=k_{s}\frac{dT}{dx}=\frac{k_{s}}{s}\left(T_{1}-T_{w}\right)$$

so that, by recalling Equation (1), the convective heat transfer coefficient may be inferred from the following relationship:

(25) $$h=\frac{k_{s}}{S}\left(\frac{T_{1}-T_{w}}{T_{w}-T_{r}}\right)-\frac{q_{r}}{T_{w}-T_{r}}$$

One of the major problems arising when the IR camera is used as a temperature transducer for the gradient sensor lies in the incapability of the camera to look at two different temperature distributions (maps), the first over the front surface and the second over the back one. This difficulty can be bypassed either by scanning the wall surfaces one at a time or by viewing both of them by means of mirrors; the first technique is more advantageous because it allows a higher spatial resolution but requires a definite steady state, the second one involves the mirror calibration.

An alternative method to make use of this sensor with IRT consists of viewing with the IR camera only the front sensor surface while keeping the back one at a given constant temperature *T_1_* (e.g., in contact with a heat sink). If *q_c_* is not relatively large, this could be achieved by exposing the back surface to a boiling liquid or a condensing vapor. The high heat transfer coefficient obtained in these conditions allows *T_1_* to coincide with the phase-change temperature of the fluid. Then in order to compensate the IR camera limited thermal accuracy (which is worse than its sensitivity), therefore eliminating the unavoidable bias, a small part of the slab could be removed to directly observe the heat sink, which emissivity however should be exactly equal to that of the sensor front surface.

### 5.2. Data Analysis, Tangential Conduction and Experimental Procedure

The gradient sensor method can also be used to a certain extent in the heat transfer unsteady regime. In particular, if the functions *T_w_*(*t*) and *T_1_*(*t*), which describe the time-temperature histories, do not contain harmonic components with frequencies greater than 0.1 *α*/*s^2^* (*α* being the slab material thermal diffusivity coefficient), the heat flux can be inferred from the formula [19]:

(26) $$q_{c}+q_{r}=k_{s}\frac{T_{1}\left(t+s^{2}/3\alpha \right)-T_{w}\left(t-s^{2}/6\alpha \right)}{s}$$

which, in particular, for *s^2^*/*α* → 0, reduces to Equation (24).

Even at steady state, the tangential conduction of this sensor cannot be longer appraised with Equation (21) that assumes a constant temperature across the slab, while in the gradient sensor, for multi-dimensional operation, a quasi-linear temperature profile exists. For a constant *T_1_* and a harmonic variation of the front surface temperature, the tangential conduction depends on the ratio **ℛ** between the wavelength of the variation and the sensor thickness *s* according to the approximate formula:

(27) $$q_{k}=-sk_{s}\nabla^{2}T_{w}/\chi$$

where the dimensionless parameter *χ* is a function of **ℛ**.

So, since it occurs that *χ* is always greater than 1, only a fraction of the Laplacian at the front side ∇^2^*T_w_* contributes to the tangential conduction along the slab.

**Figure 5.**
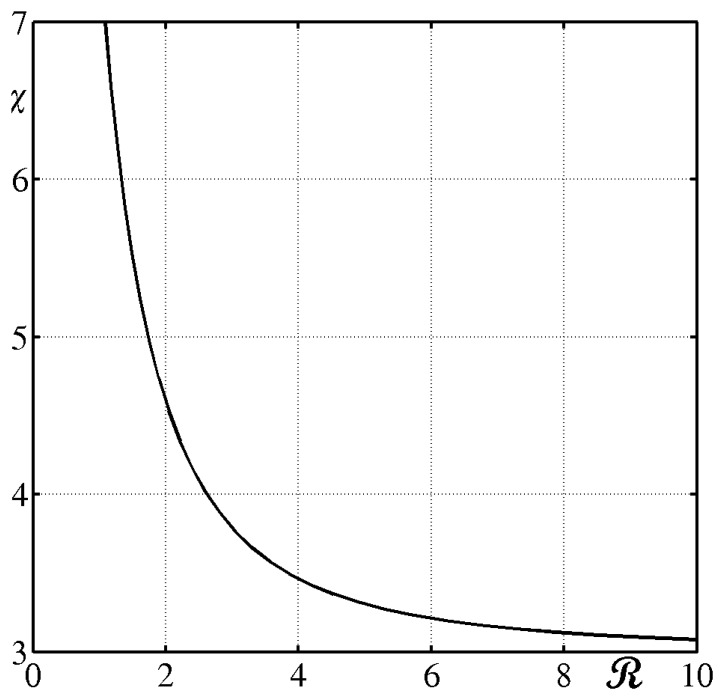
Value of χ for Equation (27) as a function of the dimensionless wavelength **ℛ**.

Values of the coefficient *χ*, computed to be used in Equation (27), are reported in Figure 5, as a function of the dimensionless wavelength **ℛ**, which shows that, at high wavelengths, only one third of the Laplacian results to be active for tangential conduction. Obviously, for more complex conditions the inverse heat transfer problem should be solved.

As far as the experimental procedures are concerned, if they apply also to the gradient sensor, the same considerations already made for the heated thin foil may be employed; e.g., when performing a steady state temperature acquisition, it is always very helpful to make an average of several temperature maps picked up in a sequence so as to reduce the random temporal noise.

## 6. Laplacian Sensor

### 6.1. Basics

Noticeably enough, the Laplacian sensor is governed by the same energy balance of the heated thin foil sensor but with a different relevance of the involved terms and the absence of Joule heating.

As already mentioned in Section 4.3, the capability of an IR scanner, to evaluate the two-dimensional distribution of the conductive heat flux, allows us to generalize the heated thin foil sensor to a more comprehensive and less complex heating condition. In particular, it is possible to heat the slab not in the measurement zone but near it and this external heating can be even non-uniform and unknown. In this way, another peculiar heat flux sensor is practically generated: the Laplacian sensor (see Carlomagno *et al.* [7]).

This (still steady state) sensor is based on Equation (23) where the term *q_j_* does not appear in the equation itself since the slab is externally heated and no Joule heating term exists in the energy balance equation within the measurement zone. In fact, the flow over the sensor surface induces the convective heat transfer that, besides the heat losses, mainly derives from the derivative of the tangential conduction heat flux within the slab, which can be appreciated by computing the two-dimensional Laplacian of the temperature distribution.

A peculiar aspect of such a sensor is therefore associated to the fact that, while for the heated thin foil sensor, *q_j_* is the main term in the energy balance equation, for this sensor, the Joule heating in the measured zone is not needed so that *q_j_* is identically zero; hence, the most important term in Equation (23) becomes indeed the derivative of the tangential conduction heat flux *s k_s_*∇^2^ *T_w_*. So it is obtained:

(28) $$h=\frac{sk_{s}\nabla^{2}T_{w}-q_{a}-{ɛ}_{t}\sigma \left(T_{w}^{4}-T_{a}^{4}\right)}{T_{w}-T_{r}}$$

Consequently, the critical feature of this sensor is the accurate evaluation of the temperature two-dimensional Laplacian because its erroneous evaluation has a direct consequence on the computation of the convective heat transfer coefficient *h*. Being dependent on the Laplacian evaluation, this heat flux sensor is also herein called *Laplacian sensor* as proposed in [7].

### 6.2. Data Analysis and Experimental Procedure

The main hindrance of the Laplacian sensor is therefore related to the importance of a correct estimation of the derivative of the tangential conduction heat flux which, with the convective one *q_c_*, are the most relevant contributions in Equation (28). As a matter of fact, being the tangential conduction usually a correction term, the heated thin foil sensor is less affected by this issue, the most important contributions arising from the convective term *q_c_* and from the imposed Joule heating *q_j_*. Therefore, because of the aforementioned reasons, for the Laplacian sensor the numerical computation of the Laplacian of Equation (28) involves some critical aspects to be considered.

An adaptive filtering technique, consisting of consecutive applications of the Wiener filter to the temperature distributions, is proposed by Rainieri *et al.* [20]. However, in the work of Carlomagno *et al.* [7], where a quite complex flow field is analyzed, a different approach is adopted, involving spatial filtering with a Gaussian window and calculating the numerical derivatives with a relatively large spatial distance.

This method implies a certain reduction of the spatial resolution of the measurement so that an operating compromise has to be necessarily found. Practically, the authors propose to correlate the dimensions of the spatial filtering window and of the numerical derivatives computational step to the physical parameters of the investigated phenomenology.

Besides, the use of an IR camera with Focal Plane Array introduces another error critical source, *i.e.*, the spatial measurement noise generated by the different t response of the several pixels of the IR detector. Therefore, an accurate and reliable process of Non-Uniformity Correction (NUC) is of fundamental importance. As a matter of fact, both spatial filtering and computation of the derivatives, with a relative large step are not often sufficient to compensate the decay of the signal-to-noise ratio due to the non-uniformities between the various pixels response.

Of course, the issue of tangential conduction correction is not herein treated because it constitutes the basic principle on which the Laplacian sensor operates.

In the application Section 9.3, the use of this sensor and its sensitivity to the heating conditions are analyzed in much more detail.

## 7. Thin Film Sensor

### 7.1. Basics

Since it measures a map of surface temperatures, the IR scanner output can be thought as originating from a two-dimensional array of an extremely large number of very small thin resistance thermometers with the advantage that the thickness of the equivalent thin films is exactly zero.

**Figure 6.**
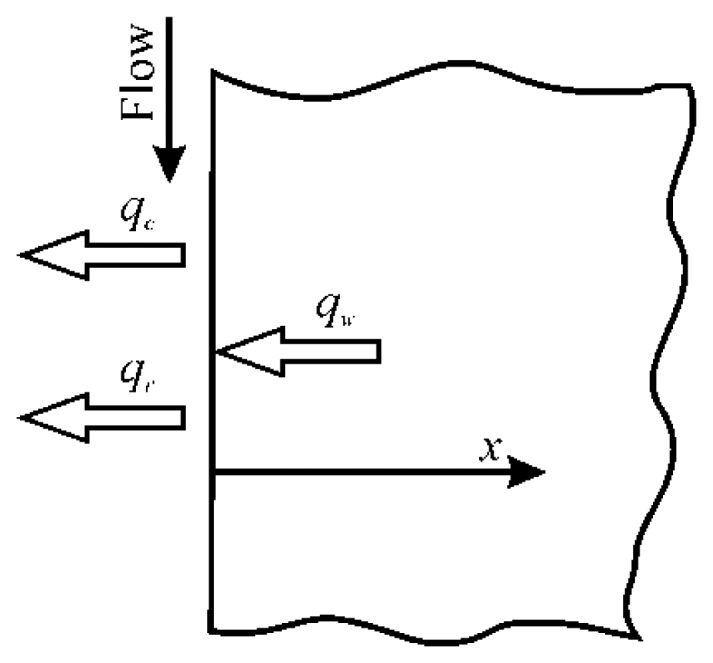
Sketch of the thin film sensor. Adapted from [1].

A very schematic sketch of the thin film sensor is depicted in Figure 6, where the temperature to be measured as a function of time *t* is *T*(*x* = 0,*t*) = *T_w_*(*t*), so that of the sensor front surface. With this sensor, since necessarily the viewed surface is that along which the fluid is flowing over, it is compulsory that the heat exchanging fluid has to be (at least partially) transparent in the IR band detected by the scanner.

It is obvious that the wall conductive heat flux within the solid *q_w_* at the fluid/solid interface, must balance the sum of the radiative *q_r_* and convective *q_c_* heat fluxes towards the fluid:

(29) $$q_{w}=q_{c}+q_{r}$$

As for the thin skin, the major part of the works that use the thin film, to measure convective heat transfer coefficients, are associated with hypersonic flows (high values of *T_r_*), so that, in Equation (1)*q_w_* becomes a negative quantity. Therefore, in the following for both this two sensors, the role of *T_r_* and *T_w_* in Equation (1) are reversed, resulting in an increase of the slab surface temperature with time.

From the classical heat conduction books (e.g., Carslaw and Jaeger [21]), it is possible to recover the one-dimensional solution for a semi-infinite wall having a constant initial temperature *T_wi_* (*x*, *t* = 0) and, at following times (*t* > 0), subjected on its surface to a uniform (in space) convective heat flux governed by Equation (1) with constant *h* and *T_r_* (which is the most commonly encountered case):

(30) $$\theta =\frac{T-T_{wi}}{T_{r}-T_{wi}}=\text{erfc}\left(\xi \right)-\exp\left(Bi_{x}-\beta^{2}\right)\text{erfc}\left(\xi +\beta \right)$$

*T* being the generic slab temperature at a certain depth *x*; the dimensionless quantities *ξ*, *β* and *Bi_x_* are defined as: $\xi =X/2\sqrt{\alpha t}$, $\beta =h\sqrt{t/\rho_{s}ck_{s}}$ and *Bi_x_* = *hx*/*k_s_*. The latter quantity represents the local Biot number, *i.e.*, the Biot number based on *x*.

In the previous notations: *α, ρ_s_, k_s_* and *c* indicate respectively the thermal diffusivity coefficient, the mass density, the thermal conductivity coefficient and the specific heat of the sensor material; *x* and *t* are respectively the spatial coordinate (starting at the interface and directed as in Figure 6) and the time variable. Often, the product *ρ_s_ck_s_* is called *thermal inertia* (or *thermal effusivity*); others use these names for the square root of the same product.

The dimensionless parameter *ξ* is proportional to the square root of the reciprocal of the local *Fourier number* (*Fo_x_* = *αt*/*x*^2^), also based on *x*. The Fourier number can be thought as measuring the relative importance of the conductive heat flux with respect to the rate of thermal energy storage but it may be certainly regarded also as a dimensionless time.

Alternatively, the dimensionless parameter *β* can be also expressed in terms of the local Biot and Fourier numbers, or *Bi_x_* and *ξ*, since:

(31) $$\beta =Bi_{x}\sqrt{Fo_{x}}=\frac{Bi_{x}}{2\xi }$$

**Figure 7.**
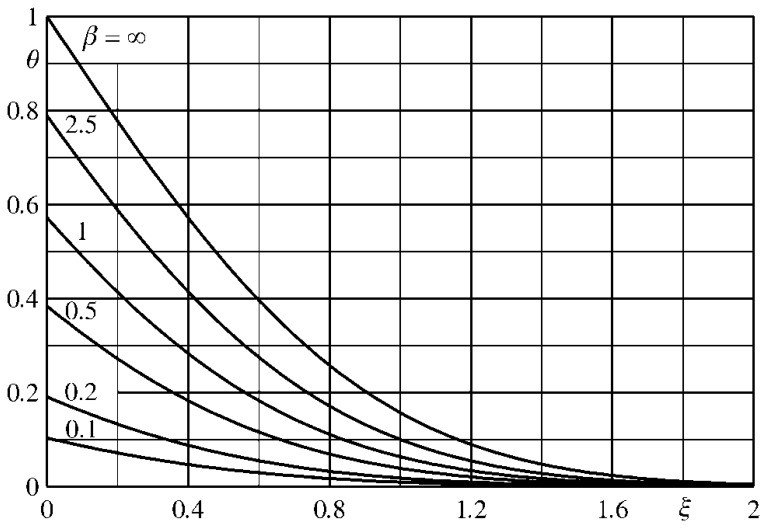
Dimensionless temperature profiles, for several *β* values, in a semi-infinite wall at a constant initial temperature and subjected to a constant *h* and *T_r_*. Adapted from [1].

The dimensionless temperature profiles into a semi-infinite wall at a constant initial temperature are plotted in Figure 7, as a function of *ξ*, for several different values of *β*.

At a given time *t*, the increase of the front surface temperature (*ξ* = 0) with *β* can be associated to either an increase of *h* or a decrease of the sensor thermal inertia *ρ_s_ck_s_*. This makes the sensor, at initial times, more sensitive if it is made of low thermal conductivity *k_s_*, and/or low thermal capacitance per unit volume *ρ_s_c*, material. On the other hand, for constant *h* and *ρ_s_ck_s_*, the increase of *θ* is connected to a time increase and, thus, to the total heat input.

The top curve of Figure 7 corresponds to *β* → ∞ (e.g., *h* → ∞) and, in this case, the boundary condition at the wall surface for *t* > 0 reduces to a constant fluid/solid interface temperature *T_w_* = *T_r_*, so that Equation (30) simplifies to:

(32) θ=erfc(ξ)

However, the case of *β* → ∞ is of no interest to measure convective heat transfer coefficients since it gives a constant temperature *T_w_* with time. This condition may be rather exploited to measure the adiabatic wall temperature *T_r_* of Equation (1), as long as the radiative contribution *q_r_* of Equation (29) can be assumed negligible.

### 7.2. Effects of the Finite Thickness of the Sensor

In practice, the thin film sensor cannot be semi-infinite but it is always made of a slab of finite thickness *s* (so also a sensor back surface does exist) and, as it can be easily understood, the equivalence to the semi-infinite wall model is valid only during a relatively small measurement time interval *t_m_*, before the thermal wave reaches the back surface. This being the case, the boundary condition on the existing back surface (e.g., *q* = 0 or *T_1_* = const) becomes practically irrelevant.

As a matter of fact, for very large values of *ξ* (e.g., large values of *x* and/or small time values), the temperature of the slab coincides with the initial one (*i.e.*, *θ* = 0), while, for *ξ* decreasing, *θ* increases.

By first supposing *β* → ∞, Equation (32) can be used to find a very conservative time limit for the correct application of the semi-infinite wall model to a finite thickness sensor.

Really, by fixing a maximum value of *θ* (for instance, 1%) which can be accepted at the sensor back surface without substantially altering the measurement, it is possible to find (for a semi-infinite sensor model) the corresponding value of *ξ* (indicated with *ξ̅*) and, therefore, the maximum measurement time that can be practically used with a finite slab [1]:

(33) $$t_{m}=\frac{s^{2}}{4{\bar{\xi }}^{2}\alpha }=\frac{s^{2}}{\alpha p}$$

where *p*=4*ξ̅*^2^ represents a constant to be computed, which is later evaluated also in less conservative limits. On a quantitative basis for *β* → ∞, by putting in Equation (32) *ξ̅* = 1.82, since *erfc*(1.82) ≈ 0.01, it is readily found *p* ≈ 13.3.

This time limit is clearly independent of the Biot number and is a very conservative one because, besides the assumption *β* → ∞ (which, as stated before, is useless to measure convective heat transfer coefficients), it considers the departure from the semi-infinite model at the sensor back surface. As a matter of fact, in the thin film sensor, the monitored temperature, which is useful to compute the heat flux, is always the front surface one.

The found time limit is in marked contrast with what affirmed by Carlomagno and de Luca [6] who suggest a *p* value equal to 2, even without specifying a back surface temperature increase as low as 1%. However, it has to be stressed that this point is often presented in the literature in a controversial way (some researchers fix *p* = 16, e.g., see Gülhan *et al.* [22]) so, in the following, the problem is examined on the condition of a *θ* accuracy at the front surface of 1%, which can represent a rather satisfactory value.

Being the slab back surface (*x* = *s*) adiabatic (which is an often encountered experimental condition) and the front one (*x* = 0) almost always subjected to a convective heat flux with constant *h* and *T_r_*, it is possible to retrieve the following exact solution [21] for the slab temperature distribution:

(34) $$\theta =1-\sum_{n=1}^{\infty }\frac{2Bi}{\cos\left(\gamma_{n}\right)\left(Bi\left(Bi+1\right)+\gamma_{n}^{2}\right)}\cos\left(\gamma_{n}\frac{1-x}{s}\right)e^{-\gamma_{n}^{2}\frac{\alpha t}{s^{2}}}$$

where now *Bi* (based on the sensor thickness) is defined as in Section 4.2 by the formula (18) and *γ_n_* are the positive roots of the equation *γtanγ* = *Bi*. Values of the first six roots of this equation can be found in [21].

Therefore, with regard to Equation (33), a less conservative measurement time limit can be found by introducing the ratio:

(35) $$\Xi =\frac{\theta_{s}-\theta_{\infty }}{\theta_{\infty }\left(0\right)}$$

which represents the percentage departure between the two solutions (for the finite slab *θ_s_*—Equation (34)—and for the semi-infinite one *θ*_∞_—Equation (30)), where the subscript *s* and ∞ refer to the finite slab and to the semi-infinite wall, respectively. The quantity Ξ can be either referred to the front sensor surface or to the back one but, as already said, only the one referring to the front surface is of interest for the thin film heat flux sensor.

The time at which Ξ is equal to a prefixed threshold (namely 0.01) can be used again as an estimate for the evaluation of the maximum measurement time. It turns out [1] that *p* is practically independent on *Bi* (the maximum value for low *Bi* being *p* ≈ 3.1). Therefore, a dependable value for *p* in Equation (29) can be definitely established at *p* ≈ 3, in most of the encountered experimental conditions.

### 7.3. Data Analysis and Experimental Procedure

In the previous sub-section it has been just assessed that, if the measurement time is sufficiently small (*t* < *s^2^*/3*α*), a slab, which is used as a thin film heat flux sensor, is well approximated by the semi-infinite wall model. Then, by supposing that the slab at the initial time (*t* = 0) is isothermal at a temperature *T_wi_*, from the front surface temperature evolution *T_w_*(*t*), measured with the infrared camera, it is possible to use the classical formula of Cook and Felderman [23] to evaluate the wall heat flux *q_w_*, as a function of time:

(36) $$q_{w}=\sqrt{\frac{\rho_{s}ck_{s}}{\pi }}\left(\frac{\varphi \left(t\right)}{\sqrt{t}}+\int_{0}^{t}\frac{\varphi \left(t\right)-\varphi \left(\tau \right)}{{\left(t-\tau \right)}^{\frac{3}{2}}}d\tau \right)$$

where *φ* (*t*) = *T_w_*(*t*)−*T_wi_* and, obviously, *q_w_* is the total conductive wall heat flux (*q_w_* = *q_c_* + *q_r_*) at the fluid/solid interface.

The previous equation is valid for a wall heat flux generally varying with time but a much simpler formula can be found when a constant wall heat flux is imposed:

(37) $$\varphi \left(t\right)=\frac{q_{w}}{2}\sqrt{\frac{t}{\pi \rho_{s}ck_{s}}}$$

where the front temperature rise is proportional to the square root of time.

Notwithstanding its simplicity, Equation (37) is of seldom met relevance in convective heat transfer measurements because, with the thin film sensor, the most commonly encountered boundary condition is of constant convective heat transfer coefficient and reference temperature in Equation (1), and not of constant wall heat flux. Anyway, a best fit, of the type later given by Equation (39), as suggested later, could be simply applied also in this case.

The solution given by Equation (37) could be considered acceptable only on condition that the quantity *φ* remains very small as compared to *T_r_*−*T_wi_*, during all the experimental test, so that the exchanged heat flux may be consequently considered almost constant.

Instead, by numerically evaluating the integral of Equation (36), it is possible to determine the wall conductive heat flux but, when using standard methods, the singularity that is present for *τ* = *t*, may reduce the results accuracy. Cook and Felderman [23] assumed that the temperature could be approximated by a piecewise linear function and found the following stable method:

(38) $$q_{w}=\sqrt{\frac{\rho_{s}ck_{s}}{\pi }}2\sum_{i=1}^{n}\frac{\varphi \left(t_{i}\right)-\varphi \left(t_{i-1}\right)}{\sqrt{t_{n}-t_{i}}+\sqrt{t_{n}-t_{i-1}}}$$

with *t_i_* = *i*Δ*t*, where Δ*t* is the time interval between the acquired data and *n* represents the total number of measured data.

Since the wall temperature history is measured with the infrared scanner, the calculation of the radiative heat flux can be made with the help of Equation (12), then, by using Equations (1) and (29), it is straightforward to evaluate also the convective heat transfer coefficient.

However, the evaluation of the conductive wall heat flux with Equation (38) is particularly suited only when a large number of measurement points in time are available and both the initial time and model temperature are well known. This is not always the case when performing tests in relatively fast transient conditions (e.g., in short duration hypersonic blow down tunnels or, in the worst instance, in shock tubes or tunnels).

In fact, in many actual unsteady measurements, it is not possible to either insert immediately the model in the main stream or to accelerate instantaneously the fluid to its final velocity value, so the initial time may be itself partially unknown.

In these circumstances, the data acquisition frequency, even of a modern IR scanner which is often of the order of 100 *Hz*, may be not large enough to accurately evaluate the wall heat flux with Equation (38).

Then, for a constant *h* and reference temperature *T_r_*, a different approach, based on a non-linear least square fit, can be more advantageous as suggested by de Luca *et al.* [24].

By writing Equation (30) at the fluid/solid interface, it is found:

(39) $$\theta_{w}=\frac{T_{w}-T_{wi}}{T_{r}-T_{wi}}=1-e^{\beta^{2}}\text{erfc}\left(\beta \right)$$

From this equation, it may be assumed that, apart from some known slab thermo-physical properties and time intervals, the convective heat transfer coefficient as well as the initial time and sensor temperature are unknown. The best fit of the measured wall temperature *T_wm_* to the exact solution described by Equation (39)*T_w_* can be found by varying *h* (in *β*) and *T_wi_* in order to minimize the following functional:

(40) $$\sum_{i=1}^{n}{\left(T_{w}-T_{wm}\right)}^{2}$$

A second problem is the radiative heat flux that, when using IR thermography, is not generally possible to neglect. Under the assumption that the convective and radiative contributions are uncoupled, de Luca *et al.* [24] propose to modify Equation (39) with the following one:

(41) $$T_{w}-T_{wi}=\left(T_{r}-T_{wi}\right)\left[1-e^{\beta^{2}}\text{erfc}\left(\beta \right)\right]-\frac{q_{r}}{h}$$

Then, by using this equation, it is possible to take easily into account also the radiative heat flux in the minimization of the Functional (36).

The previous approach is, in every respect, an inverse heat transfer problem and can be extended, by a numerical solution of the Fourier heat equation. This may be necessary in order to include the temperature dependence of the slab thermo-physical properties and/or to have, in case, slabs with high curvature (e.g., Mulcahy *et al.* [25]).

### 7.4. Tangential Conduction within the Sensor

In the previous sub-sections, also for thin film sensor, the temperature distribution within the slab is supposed to be just one-dimensional (along coordinate *x*). However, as said before, this is not the most usual case, since variations often occur over the sensor surface due to complex flow fields.

The analysis that follows is developed under the assumption that the sensor material is isotropic or, by choosing a Cartesian coordinate system with its axes directed as the two principal axes of the thermal conductivity tensor, it is always possible to split the conduction effects along the two tangential directions.

Therefore, since the extension to any arbitrary convective heat flux distribution is straightforward, for the sake of simplicity, in the following it is assumed that the convective heat flux harmonically varies only along a direction *y* parallel to the front surface of the sensor, that is:

(42) $$q_{c}=A_{q}\cos\left(\varpi y\right)$$

where *A_q_* is the heat flux amplitude and *ϖ_x_* is the wave number.

In Equation (42) it is not included any possible unsteady but uniform in space part of the convective heat flux, which is already looked upon in detail in the previous sub-sections. In fact, by considering that the involved phenomenology is linear (for an ideal sensor), the two effects can be treated separately and successively summed up.

The solution with the boundary condition (42) at the front surface and initial sensor temperature spatially constant, is given by de Felice *et al.* [26] in terms of the difference *φ* between the front surface temperature *T_w_* at time *t* and the initial (*t* = 0) sensor temperature *T_wi_* and can be put in the form:

(43) $$\varphi \left(y,t\right)=T_{w}-T_{wi}=Af\left(Fo_{\varpi }\right)\cos\left(\varpi y\right)$$

where $Fo_{\varpi }=\varpi_{x}^{2}\alpha t$ is a modified Fourier number, *A* is a constant *reference temperature amplitude* and *f* is a function of *Fo_ϖ_*. *A* and *f* are equal to:

(44) $$\begin{array}{cc} A=\frac{A_{q}}{k_{s}\varpi }; & f=\text{erf}\sqrt{Fo_{\varpi }} \end{array}$$

So, there is no phase difference (in space) between the harmonic heat flux and the front surface temperature response and *f* turns out to be an increasing function of *Fo_ϖ_* that varies between 0 and 1. Therefore, in Equation (43), *A* indicates the maximum amplitude (attained for *Fo_ϖ_*→∞) of the cosine wave while, for smaller values of *Fo_ϖ_*, the amplitudes are reduced by the *attenuation factor f*.

It has to be noticed that, despite the fact that the attenuation factor is an increasing functions of the Fourier number, the effective temperature amplitudes *Af* increase for decreasing spatial frequencies and the opposite is true for *ϖ_x_* → 0. In particular, in this latter case, the effective amplitude limit is:

(45) $$\underset{\varpi \to 0}{\lim}\frac{Af}{A_{q}}=\frac{2}{\sqrt{\pi }}\frac{\sqrt{\alpha t}}{k_{s}}=\frac{2}{\sqrt{\pi }}\sqrt{\frac{t}{\rho_{s}ck_{s}}}$$

As expected, the limits is unbounded for increasing *t* since the problem is reduced to a constant (in time and space) boundary condition.

To correct the measured temperatures so as to take into account tangential conduction effects, it is convenient to evaluate the ratio between the effective temperature amplitude *A f*(*Fo_ϖ_*) (as computed from Equation (44)) and that corresponding to the same value of *A_q_* but in absence of tangential conduction (*i.e.*, that given by the one-dimensional solution or, analogously, by the limit of Equation (45).

By defining this ratio as the *temperature modulation transfer function F*, it results:

(46) $$F=\frac{\sqrt{\pi }}{2}\frac{\text{erf}\left(\sqrt{Fo_{\varpi }}\right)}{\sqrt{Fo_{\varpi }}}$$

**Figure 8.**
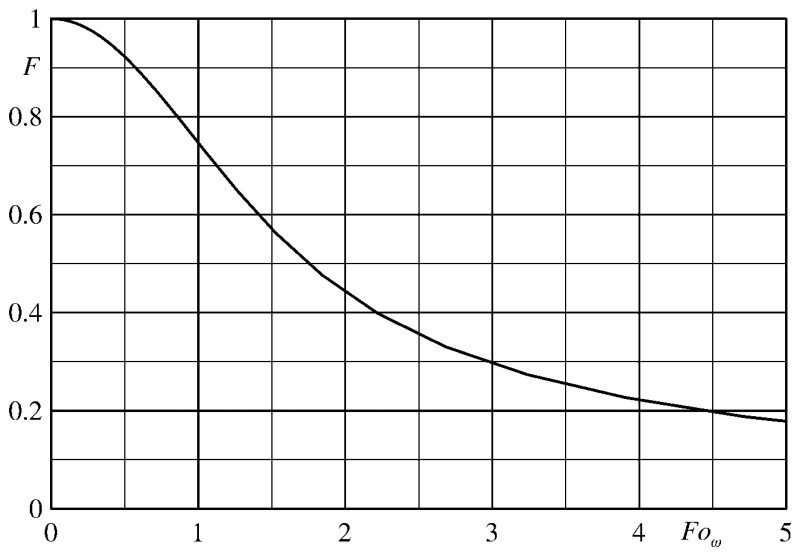
Temperature modulation transfer function *F* for the thin film sensor. Adapted from [1].

Once the function *F* represented in Figure 8 is known, the amplitude of each harmonic component of the measured temperature may be corrected and the restored temperature maps can be used to compute the effective heat flux by using the formulae already presented in Section 7.3.

As a matter of fact, it turns out that, for the tangential conduction in a thin film sensor, there is a kind of modulation function in time (*Fo_ϖ_*) and not one in space as it happens for the modulation transfer function that occurs for the scanner spatial resolution [1].

An example of image restoration for the thin film sensor is reported in Section 9.2.

## 8. Thin Skin Sensor

### 8.1. Basics

**Figure 9.**
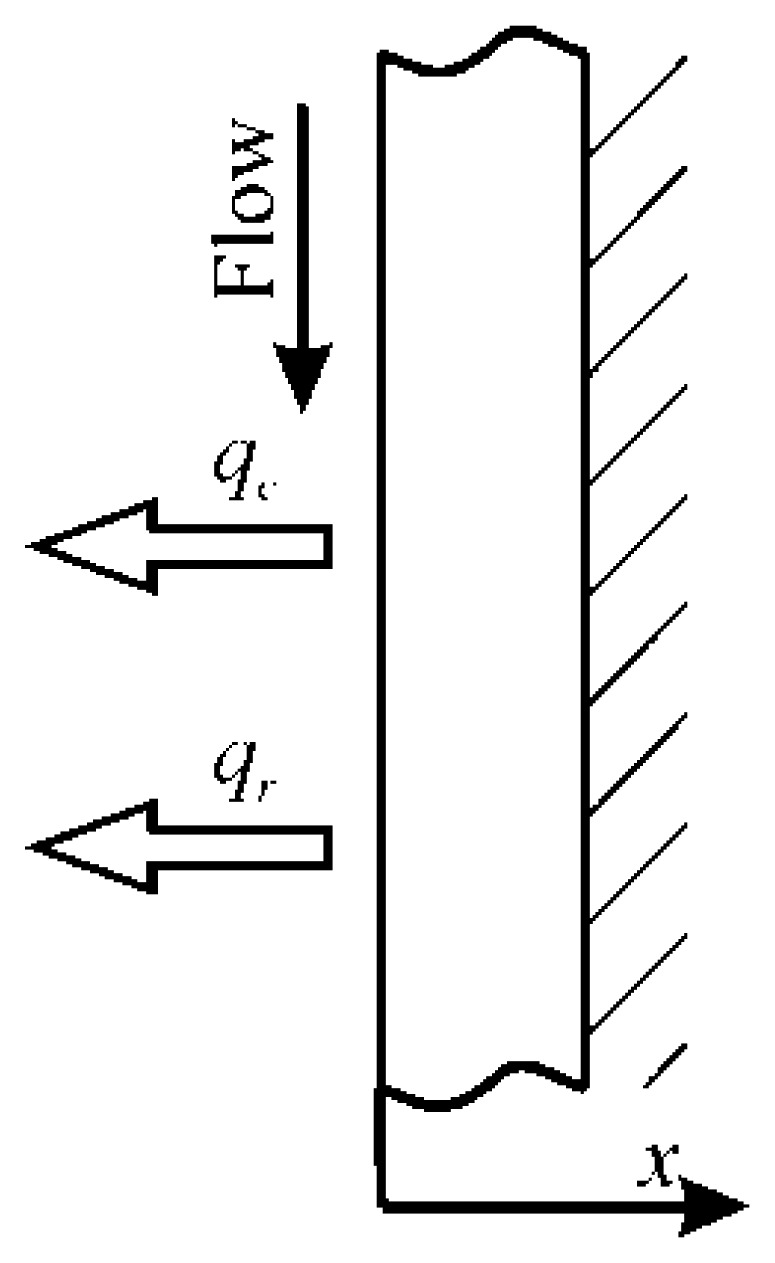
Sketch of the thin skin sensor.

In the case of the thin skin (or wall calorimeter), the sensor, practically a thin slab (see the sketch of Figure 9) of thickness *s*, is usually modelled as a perfect calorimeter (isothermal across its thickness, so thermally thin) which is heated at the front surface and thermally insulated at the back one.

As it will be shown later, the isothermal condition involves that the slab has to be not only thin but also with a high thermal conductivity coefficient as it happens, e.g., for metals.

Even if the isothermal condition is not fulfilled, the unsteady one-dimensional energy balance applied to the slab is:

(47) $$q_{w}=-\rho_{s}cs\frac{dT_{m}}{dt}$$

where *T_m_* is the mean temperature across the slab thickness and *q_w_* is the external heating which must always fulfil the condition *q_w_* = *q_c_* + *q_r_*. In the following sub-sections, *Bi* and *Fo* are always based on the slab thickness *s*.

Having assumed the slab isothermal (which as it will be seen is true as long as *Bi* ≪ 1 and for relatively large *Fo* values), the wall heat flux *q*_w_ and consequently the convective heat flux can be quite easily computed by numerically evaluating the time derivative of the temperature measured on either side of the sensor.

If the back surface of the sensor is not adiabatic for measurement needs, Equation (47) can be extended, as already discussed for the heated thin foil sensor, by including the total heat flux *q*_a_ to the ambient environment.

When the imposed wall heat flux is constant with time and the sensor is ideal, Equation (47) can be easily integrated and the result is practically a linear increase of the slab mean temperature with time. In particular, in this case, as in the following one, it is assumed that the temperature rise is the same across the slab thickness.

As shown later and apart from the influence of the radiative heat flux, an essentially exponential rise of the temperature with time can be easily found when a convective heat flux, with constant *h* and reference temperature *T_r_*, is enforced at the wall (see Equation (51) in the following).

However, in both the above described cases, the simple steady state solution is preceded by a transient (for low *Fo* values), where the temperature at every slab point rises from its initial value to an asymptotic dependence. As it will be seen in the next section, during this transient, the rate of variation of the wall temperature (on both sides of the sensor but especially at the front one) can be significantly different from the mean one.

Furthermore, it has to be stressed that it is not generally convenient to directly evaluate either heat fluxes or convective heat transfer coefficients with derivatives of acquired temperatures because the measured experimental signal is unavoidably affected by noise.

Some solutions to overcome this problem are to either previously filtering the signal or adopting an integral approach or else minimize a functional of the type given by Equation (40).

### 8.2. Isothermal Assumption

There are many experimental circumstances where the testing time is relatively short, such as short running time blow down wind tunnels or shock tunnels, and the limits of the thin skin sensor isothermal conditions have to be correctly assessed.

The isothermal condition of the thin skin can be expressed by introducing the dimensionless parameter:

(48) $$\Theta =\frac{\theta_{w}-\theta_{1}}{1-\theta_{w}}$$

where *θ* has the same definition given in Section 7.1 and the subscripts *w* and 1 again respectively indicate the front and back surfaces of the sensor. As it will be seen later, the condition |Θ| ≪ 1 is realized, also at the asymptotic state (*i.e.*, relatively large *Fo* values), as long as the Biot number is sufficiently small.

**Figure 10.**
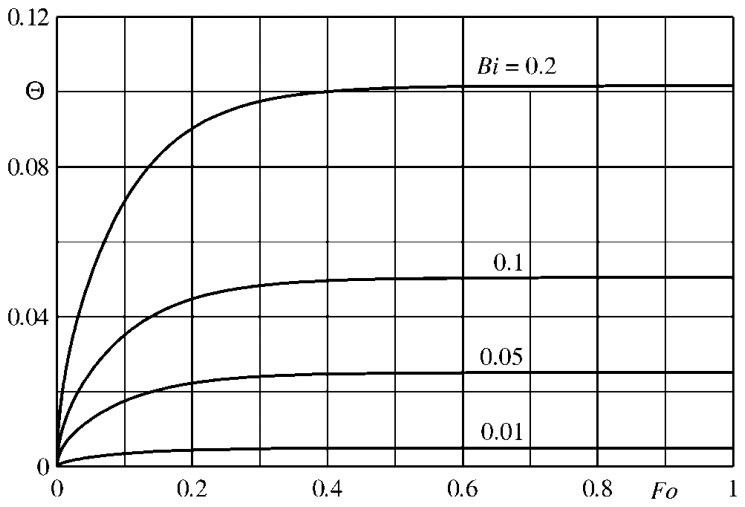
Θ as a function of *Fo* for several *Bi* values. Adapted from [1].

By supposing again the slab adiabatic at its back surface and subjected to a convective heat flux, with constant *h* and *T_r_*, and by using Equation (34), in Figure 10 the parameter Θ is plotted as a function of *Fo* for different values of the Biot number. Initially, the temperature is constant inside the slab so the parameter Θ is small regardless of the Biot number. Afterwards, Θ increases and reaches an asymptotic value, which is practically attained for *Fo* > 0.5 and appears to be an almost linear function of *Bi*, as it should be expected.

**Figure 11.**
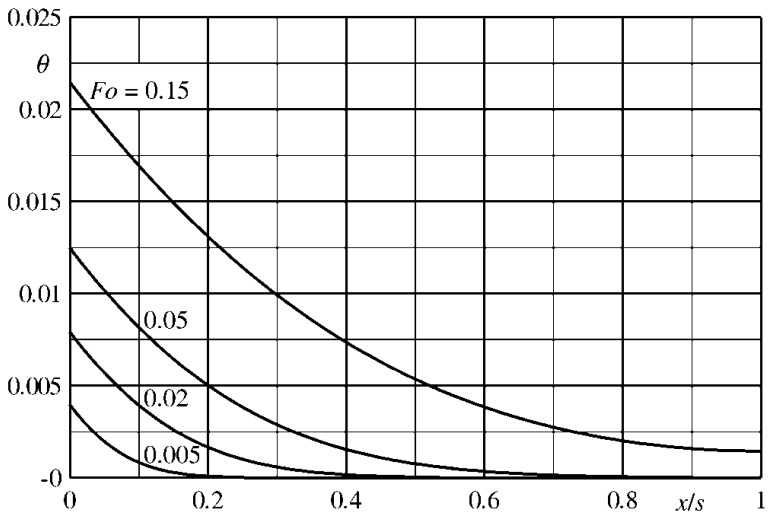
Temperature profiles in the slab (*Bi* = 0.05) at several *Fo* values for adiabatic back surface.

The isothermal condition, previously enforced, does not imply that, as shown in Figure 11, the temperature across the slab, even for a quite small Biot number (e.g., *Bi* = 0.05), is practically constant. Furthermore, for very small *Fo* values, the thermal wave is not able to yet reach the opposite wall (sensor back surface).

Clearly, if the IR scanner measures the back temperature *T_1_*, the time derivative of the measured temperature is, initially, equal to zero, thus it is necessary to wait some time before acquiring the measurement.

On the contrary, the sensor front surface temperature initially increases more rapidly than the average one and, for small Fourier numbers, also this temperature evolution does not enable to correctly evaluate the time derivative of the mean slab temperature (which is the one that monitors the heat flux, see Equation (47)).

The error made in the evaluation of the mean temperature time derivative can be expressed in a quantitative way with the dimensionless ratio:

(49) $$\Pi =\left(\frac{dT}{dt}-\frac{dT_{m}}{dt}\right)/\frac{dT_{m}}{dt}$$

which is a function of the position in the slab and of the Fourier and Biot numbers.

**Figure 12.**
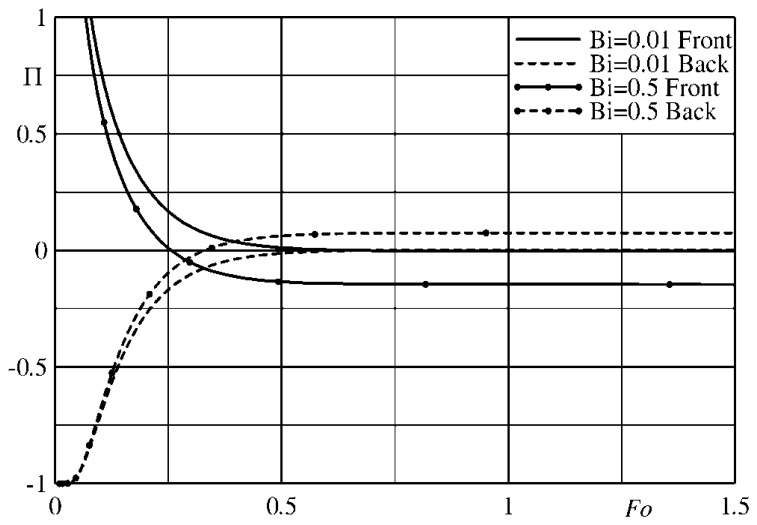
Error in the evaluation of the derivative of the mean slab temperature. Adapted from [1].

In Figure 12 for both the front and back surfaces, the ratio Π is plotted as a function of the Fourier number for two *Bi* values. For very small times (*i.e.*, *Fo*), at the back surface the condition dT/dx=0 occurs and this event explains the initial 100% constant value of the negative error. On the contrary, at the front surface the temperature variation is much larger than the mean one and this is the cause for the extremely large positive error at very low *Fo* values.

From the graph, it is evident that both absolute errors decrease for increasing *Fo*; they become smaller than 1%, for *Bi* = 0.01 and Fourier numbers larger than 0.5. The error curves remain practically unchanged as long as *Bi* < 0.05 while, as it can be noticed from the figure, for the larger Biot number the asymptotic error increases significantly but the time needed to reach its final value remains practically the same.

It is interesting to notice that, for the high Biot number, the asymptotic error for the back surface is smaller (about 0.075) than that for the front one (about −0.15) and this because its temperature is always closer to the mean one (e.g., see Figure 11).

At the asymptotic state and when the Biot number is not appropriately small, it is still possible to use the thin skin sensor but it is necessary to adopt a more accurate approximation of the mean slab temperature or, as it is shown in the following sub-section, of the time at which evaluating it from the measured data.

### 8.3. Data Analysis and Experimental Procedure

The computation of the convective heat transfer coefficient can be performed by rearranging Equations (1), (25) and (47) so as to obtain [1]:

(50) $$h=\frac{-\rho_{s}cs\frac{dT_{m}}{dt}-q_{r}}{T_{w}-T_{r}}$$

For quite small Biot numbers and *Fo* > 0.5, the slab can be considered as isothermal and it is possible to evaluate *T_m_* with either the front *T_w_* or the back *T_1_* surface temperature. The solution of Equation (50) for a constant convective heat transfer coefficient and for *q_r_* = 0 (but a similar result is also obtained, whenever possible, by linearizing *q_r_* and adding to *h* the radiative heat transfer coefficient *h_r_*) is given by:

(51) $$\frac{T_{w}-T_{r}}{T_{wi}-T_{r}}=\exp\left(-\frac{ht}{\rho_{s}cs}\right)$$

This relation can be used to implement a regression process to measure *h* as in the case of the thin film previously described.

A different approach is based on the numerical solution of Equation (50); a simple central difference formula may be normally sufficient:

(52) $${\left(\frac{dT}{dt}\right)}_{i}=\frac{T\left(t_{i+1}\right)-T\left(t_{i-1}\right)}{2\Delta t}$$

where the time interval is defined as 2Δ*t* = *t_i_*_+1_ − *t_i−_*_1_.

However, as said above it is advisable, before using Equation (52), to filter (in time) the temperature signal to avoid errors due to the captured noise.

As previously mentioned, when the slab cannot be considered as isothermal (*Bi* > 0.05), or the measurement is unsteady (as it may happen because of the continuous change of *T_w_* in Equation (1) and the non-linear change of *q_r_*), approximating the mean temperature with that of one of the surfaces may lead to significant errors in the evaluation of the wall heat flux.

In the following, some formulae, which are useful to reduce unsteady convective heat transfer measurements with the thin skin sensor (such as the case of constant *h* and *T_r_*), are reported.

By making use of the Laplace transform and considering a first order expansion, it is possible to find the following approximate relations (Douglas [19]) between the front surface temperature *T_w_*, the mean *T_m_* and the back surface one *T_1_* (the right insulated surface of Figure 9):

(53) $$T_{w}\left(t-\frac{s^{2}}{3\alpha }\right)≅T_{m}≅T_{1}\left(t+\frac{s^{2}}{6\alpha }\right)$$

where *s* is the sensor thickness and *α* its thermal diffusivity coefficient.

By numerically differentiating Equation (53), a better estimate of the mean temperature derivative can be easily accomplished for unsteady measurements. In particular, if the IR scanner measures the back surface temperature, the use of Equation (53) enables to link easily *T_1_* to *T_m_* and the front surface temperature *T_w_* needed in Equation (50) to evaluate the convective heat transfer coefficient.

In the conventional heat flux measurement techniques, the temperature is normally measured with a thermocouple bonded to the rear of the sensor (the back surface) and higher accuracy reduction methods are reported. The main idea is to use a Taylor expansion (truncated at the order 2*n*) of the temperature distribution inside the slab and to exploit the adiabatic boundary condition and the Fourier equation so as to obtain:

(54) $$T\left(x\right)≅T_{1}+\sum_{i=1}^{n}\frac{d^{2i}T_{1}}{dx^{2i}}\frac{{\left(x-s\right)}^{2i}}{\left(2i\right)!}=T_{1}+\sum_{i=1}^{n}\frac{d^{i}T_{1}}{dt^{i}}\frac{{\left(x-s\right)}^{2i}}{\alpha^{i}\left(2i\right)!}$$

where the coordinate *x* is oriented as in Figure 9 and the second equality derives from the Fourier's equation.

Equation (54), where the dependence from *t* has been dropped for sake of ease, can be used (by simply imposing *x* = 0) to evaluate the temperature *T_w_* on the opposite side of the slab.

Jepps [27] proposes the following second order discretisation:

(55) $$T_{w}\left(t\right)≅3T_{1}\left(t+\frac{s^{2}}{6\alpha }\right)-2T_{1}\left(t\right)$$

By integrating Equation (54) in space, the result can be used to compute also the mean temperature:

(56) $$T_{m}≅T_{1}+\sum_{i=1}^{n}\frac{d^{i}T_{1}}{dt^{i}}\frac{s^{2i}}{\alpha^{i}\left(2i+1\right)!}$$

By differentiating in time the previous equation, one obtains the following relation that, for unsteady measurements, extends to higher frequencies the field of validity of the thin skin sensor:

(57) $$\frac{dT_{m}}{dt}≅\sum_{i=0}^{n}\frac{d^{i+1}T_{1}}{dt^{i+1}}\frac{s^{2i}}{\alpha^{i}\left(2i+1\right)!}$$

In the first order approximation (*n* = 0), the previous formula obviously reduces to the case of an isothermal slab, while for higher orders, the time derivatives can be easily numerically evaluated.

For *n* = 1 Jepps [27] proposes the following discretisation (that is equivalent to a first order numerical approximation of Equation (53)):

(58) $$\frac{dT_{m}}{dt}≅\frac{3\alpha }{s^{2}}\left(T_{1}\left(t+\frac{s^{2}}{3\alpha }\right)-T_{1}\left(t\right)\right)$$

Generally, higher order approximations are not required but, should it be necessary, they can be easily implemented from (57).

As already said, since with conventional techniques the temperature that is measured in wall calorimeters is generally the back surface one (e.g., with a leaf-type thermocouple), in the classical literature there is less interest in formulae based on the front surface temperature. Instead, the latter is helpful with IR thermography where *T_w_* can be easily measured when dealing with gas flows.

Nevertheless, a formula equivalent to Equation (54) can be found by again imposing the adiabatic condition on the back surface:

(59) $$T\left(x\right)≅T_{w}+\sum_{i=1}^{n}b_{i}\frac{d^{i}T_{w}}{dt^{i}}\frac{x^{2i}}{\alpha^{i}}$$

where the first five coefficients *b_i_* are equal to 1, −1/3, 2/15, −17/315, 62/2835. Again, Equation (59) can be used directly (by simply imposing *x* = *s*) to evaluate the temperature on the back sensor surface or, after integrating in space, to compute the mean temperature and its derivative with numerical formulae.

### 8.4. Tangential Conduction

A solution in the form of (43) has been also developed by de Felice *et al.* [26] (see [11]). It has, however, to be explicitly noted that, when the temporal variations are not too fast, the tangential conduction correction for the thin skin sensor can be more directly appreciated as for the case of the heated thin foil with Equation (21) but, in this case, as a function of time.

## 9. Applications

The applications of IR thermography to thermo-fluid-dynamics encompass a very much diversified phenomenology which spans from turbine cooling including film cooling, to transition separation and reattachment, natural and forced convection, enhanced heat transfer, micro systems, rotating bodies, impinging jets, flow instabilities, two-phase and hypersonic flows, *etc*.; a review is presented in [1].

The authors' research group has been involved in IR thermography since about 30 years. In the following, a few significant papers of this group, essentially regarding complex fluid flows and specifically investigated with IR thermography, are presented and reviewed, also with the aim of pointing out in many of them a few relevant aspects of the used sensors.

### 9.1. Natural Convection

When studying with IR thermography natural convection in fluids transparent to infrared radiation (such as in gas flows), since the radiative heat flux may be often of the same order of magnitude of the convective one, this occurrence has to be carefully considered. For example, when determining the convective heat transfer coefficient between the sensor and a gas with the heated thin foil sensor, the substantial radiative contribution must be accurately subtracted to the Joule heat input (see Section 7.1).

The experimental apparatus, used by Cardone and Carlomagno [9], to analyze transient and steady one-dimensional natural convection on a vertical plate with the heated thin foil, is shown in Figure 2 and also reported in [28].

The sensor is made of a vertical stainless steel foil (245 mm high, 960 mm wide and 40 μm thick), coated on one side with a thin layer of high emissivity (*ɛ_t_* = 0.95) paint. The foil is Joule heated with a DC stabilized power supply and a step initial condition can be imposed by activating a relay with a relatively small (with respect to the foil characteristic time) activation time. The reached heat flux is varied in the range 80 ÷ 270 W/m^2^. By measuring apparent temperatures at both foil sides, the uncoated side emissivity is evaluated to correct for radiation losses also from this side.

**Figure 13.**
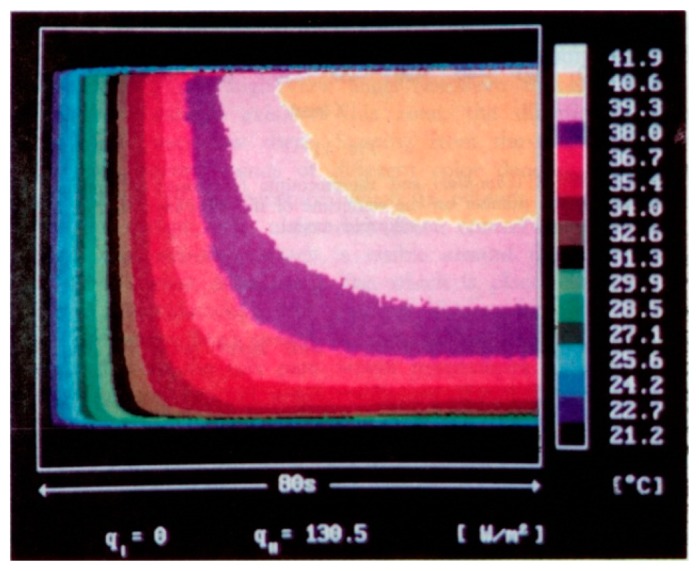
Time evolution of the temperature vertical profile on the foil under transient natural convection. From Carlomagno and de Luca [28].

For a step Joule heat flux input 0 → 130.5 W/m^2^, the evolution with time of the temperature vertical profile along the foil central segment is shown in the pseudo-thermogram of Figure 13 where: abscissa is time (starting from the relay activation and for a total of 80 s), ordinate is position along the foil central vertical segment (0 ÷ 245 mm) and colors indicate local temperatures (21 ÷ 42 °C).

The thermogram left region, characterized by a sequence of colored vertical bands, points out an initial uniform time increasing of foil temperature which indicates that the foil is acting essentially as a thin skin sensor with respect to the Joule input, Equation (47) with *q_w_* = *q_j_ − q_r_*. Besides, this means also that a prevalent radiative and conductive heat transfer regime is initially established between the foil and the ambient air that produces a constant temperature along the vertical direction; so, it appears that the convective flow is not started yet. This initial foil temperature evolution well agrees with theoretical calculations which include conduction and radiation from the foil to ambient.

**Figure 14.**
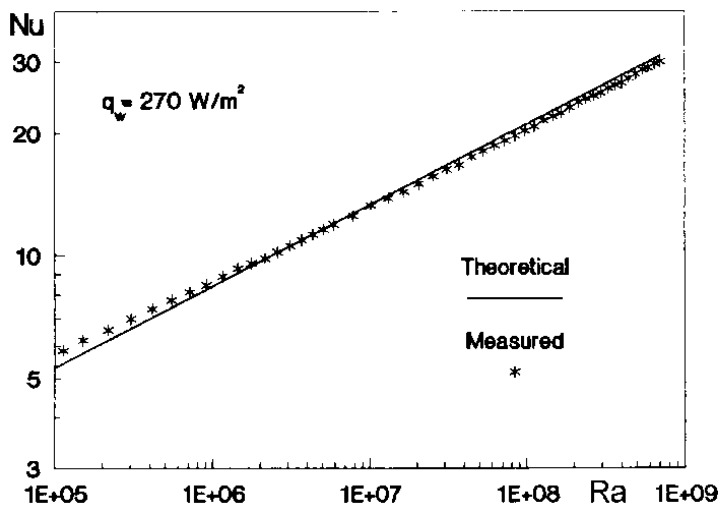
Nusselt number on a vertical plate cooled by natural convection as a function of the Rayleigh number. From Carlomagno and de Luca [28].

The temperature variation which later occurs along the vertical direction shows the progressive onset of the natural convection boundary layer. When steady state is reached, Equation (14) applies and, as represented in Figure 14, where local *Nu* data are plotted as a function of the local modified Rayleigh number *Ra* defined in the standard way, the measured data well agrees (within ±5%) with the theoretical prediction of Sparrow and Gregg [29] for laminar flow.

Obviously, all data is corrected for the radiative contribution, that is subtracted to the Joule input as a function of position, but not for tangential conduction, which mainly affects the low *Ra* data.

### 9.2. Hypersonic Flow over a Ramp

A relatively old example of the restoration of a degraded image, which considers both the IR system modulation transfer function (MTF) and the tangential conduction in the thin film, is reported by de Luca *et al.* [30]. They perform heat transfer tests on a ramp which follows a delta wing in a hypersonic wind tunnel. Since heat transfer measurements are performed with the thin film sensor, after starting of the tunnel, the model (which is initially in a remote position at room temperature) is suddenly injected into the high stagnation temperature (800 K) hypersonic stream and its temperature monitored.

**Figure 15.**
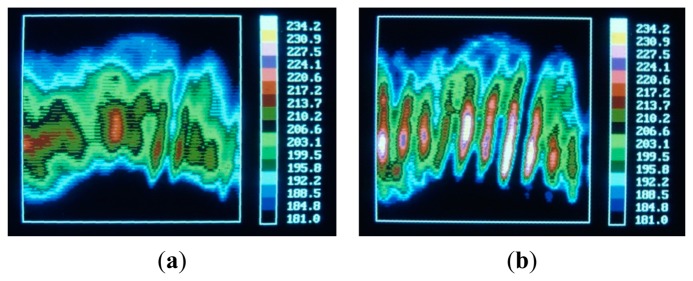
Images of Görtler vortices on a 15° ramp placed downstream of a 70° delta wing in a hypersonic flow at *M* = 8.15: (**a**) coarse; (**b**) restored. Flow from bottom to top. From de Luca *et al.* [30].

The gas flows over a ramp, placed downstream of a 70° delta wing at zero angle of attack, at Mach number *M* = 8.15. Figure 15a shows a detected thermogram with the coarse footprints (*T* in °C) of the Görtler vortices on the solid 15° RTV elastomer ramp as recorded by the camera. The Görtler vortices develop on the ramp reattachment zone due to the streamlines curvature induced by the ramp presence (flow going from the thermogram bottom, hinge line, upwards). In the raw thermogram of Figure 15a, the vortices are barely evident in the span wise quasi-periodic horizontal variation of wall temperature.

The amplitude of each harmonic component of measured temperatures has been corrected for the MTF and by means of the function *F* (see Equation (46). The restored temperature map, that shows the effective temperatures, is represented in Figure 15b. It has to be observed that, even if the used interlaced 880LW camera (AGEMA, Sweden) has a limited spatial resolution (140 × 140 pixels), being operated with (36 + 20) mm extension rings at the minimum focus distance (with a 21 mm × 21 mm field of view), the image is well sampled so degradation is due to camera and sensor. Anyhow, the restored image allows us to correctly identify the rather regular Görtler vortices structure which exhibits a mean pitch of approximately 2 mm.

The horizontal striping, which is evident in the vortices prints for both the coarse and restored thermograms, is exclusively due to the camera interlacing and to the transient heating of the ramp that is connected to the unsteady thin film sensor functioning mode. In their work, de Luca *et al.* [30] address also the problem of the signal digital sampling, which is not herein analyzed. A similar investigation has been performed by de la Chevalerie *et al.* [31].

De Luca *et al.* [32] report flow visualizations and convective heat transfer measurements on a double ellipsoid model in a hypersonic stream at *M* = 8.15 and several angles of attack *α*. To obtain heat transfer data, the models are made of NORCOAT^®^ 4000 (Norco Industries, Compton, CA, USA) which is a silicon elastomer filled with hollow silica microspheres that has a relatively low thermal conductivity coefficient (*k* = 0.129 W/m K) and a high emissivity coefficient (*ɛ_t_* = 0.93). Besides performing standard oil film visualizations and in order to exploit the capability of the infrared camera to detect an induced transition, several trip cylindrical wires (0.5 mm high, 0.22 mm in diameter and placed at 5 mm steps) are implanted 30 mm away from the ellipsoid nose, on the windward surface, with their axes normal to this surface.

**Figure 16.**
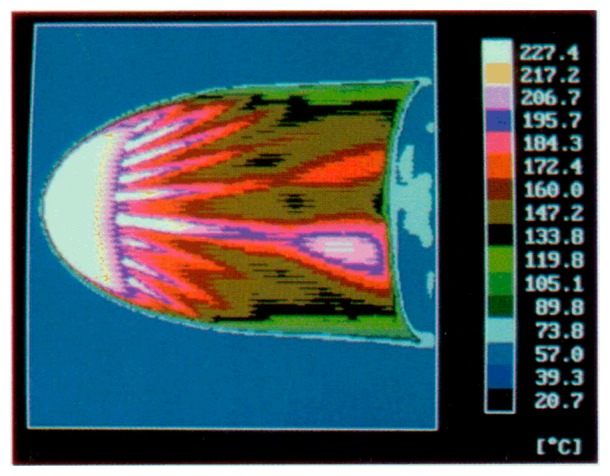
Temperature (in Celsius) map of the ellipsoid side (windward) of the double ellipsoid model at M = 8.15, *α* = 30°, 0.48 s after model injection. Flow from left to right. From de Luca *et al.* [32].

The thermogram of Figure 16, where temperatures are in Celsius, shows the effect produced by the trip wires on the temperature distribution over the model windward surface, 0.48 s after model injection. To better distinguish the flow behavior downstream of the trip wires, the temperature range of the used 880LW camera (AGEMA, Sweden) is set so as to have the ellipsoid forepart in saturated conditions.

The wakes of the wires are made evident by the various stripes, which indicate the surface flow streamlines direction over the model surface. They start downstream of the transversal violet line of the thermogram, where the transition trips are approximately located. In the model lateral zones, where the fluid particles following curved divergent path lines accelerate, the turbulisation induced to the flow by the trips seems to damp out, denoting a flow re-laminarization. On the contrary, near the symmetry axis, the flow after re-laminarization becomes downstream unstable and the occurrence of turbulence seems evident in the two hot red and pink spots located close to the model trailing edge.

Unlike standard oil film visualizations, this procedure does not need to have the model surface sullied and renovated after each test; e.g., if the angle of attack has to be changed, it is only necessary to retrieve the model back in the remote position and let it recover a uniform temperature.

### 9.3. Jet Impinging on a Plate

The group of Carlomagno has extensively studied jets impinging on a plate with IR Thermography [4,6,7,28,33–35]; herein first, the measurement of the convective heat transfer coefficient by means of the Laplacian sensor [7] is presented, then the adiabatic wall temperature detection [4] is treated.

The first tackled application task is the measurement of the distribution of the convective heat transfer coefficient over a thermally thin plate subjected to a jet normally impinging on it, at a short nozzle-to-plate distance, which originates a quite known but rather complex thermal field.

The experimental procedure and apparatus are the same used by Meola *et al.* [4]. The main difference is that the impinging sensor plate is no longer a heated thin foil as in [4] but rather an externally heated 1.1 mm thick metal sheet horizontally placed, with the jet normally impinging underneath, in order to minimize the effects of hot air recirculation in the measurement zone.

An aluminum alloy (Al–3105, *k* = 185 W/mK) hexagonal plate, constitutes the impinging target which has a free circumscribed circle about 300 mm in diameter. To correctly evaluate tangential conduction, both thickness and thermal conductivity of the sensor plate are precisely known.

**Figure 17.**
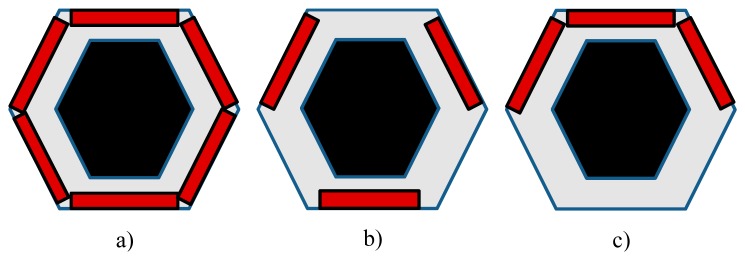
Schematic configurations of the peripheral heaters in the plan view of the plate: (**a**) Hexagonal; (**b**) Triangular; (**c**) One-side. From Carlomagno *et al.* [7].

The heat input is provided by means of electric cartridge heaters, placed inside copper bars, which are positioned at the plate sides. In order to investigate the sensitivity of the sensor to the heating geometry, three different peripheral positions of the external heaters are tested (see Figure 17): *hexagonal* (six heathers on all hexagon sides, Figure 17a), *triangular* (three heathers on each other side, Figure 17b) and *one-side* (three heaters placed on three adjacent sides, Figure 17c). The black-painted hexagonal zones of Figure 17 coincide with the measurement region. In order to reduce the total radiative heat flux towards the ambient, only these zones observed by the IR camera are covered with black paint.

Even if compared with the relatively high local convective heat transfer coefficient, the rather small thermal thickness of the aluminum plate determines a very small Biot number. Consequently, the temperature can be considered as practically constant across the plate, so allowing for measuring the surface temperature distribution with the IR camera by observing the sensor back surface, *i.e.*, that opposite to the jet impinging one. An infrared camera QWIP FLIR SC6000 LW (640 × 512 pixels resolution) operating in the LWIR band is employed to measure the surface temperature map.

The jet issues from a slightly convergent axisymmetric nozzle with an exit diameter *D* = 18.7 mm. The Reynolds number *Re* = 30,000 is based on the nozzle diameter *D* and jet initial velocity. The convective heat transfer coefficients are presented in terms of Nusselt number (2), also based on *D*.

Tests are carried out by varying the dimensionless nozzle-to-plate distance *z*/*D* but herein only results for *z*/*D* = 2 are reported, being them the most intriguing ones in terms of *Nu* radial profile. For each test, the heat input is chosen by trying to maintain on the slab a minimum temperature difference of at least 15 K with respect to the jet temperature. Obviously, as shown by Equation (28), the higher the minimum temperature difference, the more accurate the results while evaluating *h*.

Anyhow, a limit is imposed on the maximum operative temperature of the plate; e.g., restriction are imposed by the camera calibration range, the maximum heat input of the heating elements and, overall, the melting point of the black paint. Since in the performed experiments the calibration of the IR camera system is made between 15 and 80 °C, the heaters are shielded with insulated material in order to avoid saturated pixel values.

The physical and the geometrical parameters of the experimental apparatus are carefully chosen. E.g., the distance between the heaters, the slab thermal thickness and the outflow conditions directly influence the distribution of the convective heat transfer and, consequently, a suitable temperature distribution for the Laplacian numerical computation. For these reasons, it is suggested that an accurate preliminary study of the phenomenology has to forego the application of this sensor.

First, the effect of different sources of measurement noise, affecting the Laplacian computation, is investigated. Results show that the temporal noise can be consistently reduced if each temperature map is obtained by averaging a relatively high number of images. In the present investigation, 100 instantaneous images are averaged to obtain each steady-state temperature map.

Then as already stated, the use of an IR camera with Focal Plane Array introduces another error critical source, *i.e.*, the spatial measurement noise generated by differences in the response of each detector pixel. Therefore, the application of an accurate and reliable process of Non-Uniformity Correction (NUC) is of fundamental importance. In fact, tests demonstrate that both spatial filtering and computation of the derivatives with a relative large step are not sufficient to compensate the decay of the signal-to-noise ratio due to the non-uniformities between the various pixels response. Carlomagno *et al.* [7] show that even a small local error (of the order of 0.1 K) can completely jeopardize the accuracy of the computed Nusselt number distribution in a region whose dimension is connected to both the spatial filtering window and the step for the derivatives numerical computation.

**Figure 18.**
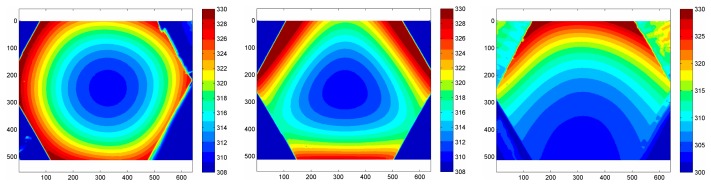
Steady state temperature (K) of the plate without the jet. From left to right: hexagonal heating configuration; triangular; one-side. From Carlomagno *et al.* [7].

In Figure 18, the steady state temperature maps for the three configurations without the impinging jet are reported. As previously stated, the slab sides are shielded in order to avoid saturated pixel values in the viewed scene. The temperature distributions are convolved with a 30 × 30 pixels wide (about 0.5*D* × 0.5*D*) Gaussian filtering window, with a standard deviation equal to 6. From the temperature maps, it is evident that a more regular distribution occurs for the hexagonal heating configuration that allows us to better evaluate the convective heat transfer coefficient from the temperature Laplacian.

Further increase of the signal-to-noise ratio influence is recovered by computing the numerical derivatives with a spatial step of one nozzle diameter.

**Figure 19.**
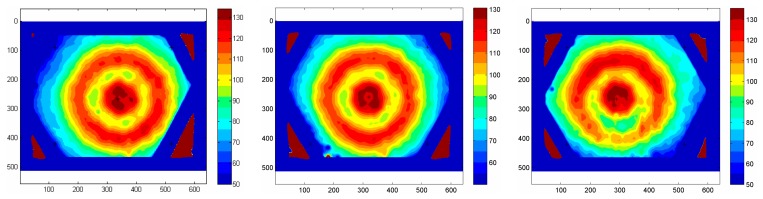
Nusselt number distribution for *Re* = 30,000 and *z*/*D* = 2. From left to right: hexagonal heating configuration; triangular; one-side. From Carlomagno *et al.* [7].

In Figure 19, the computed Nusselt number distributions for the three tested configurations are shown. The distributions seem to be quite independent of the heaters arrangement (see the figures relative to the hexagonal and triangular configurations), unless the strongly asymmetric heating (one-side) is adopted. In this latter case, it is clear that, since the condition of a maximum temperature is imposed, the minimum temperature is lower than in the case of more symmetric configurations (see the bottom zone of the right image of Figure 18). As can be seen, this decreases the tangential heat transfer and accordingly increases the uncertainty due to the lower difference between wall and jet temperatures.

**Figure 20.**
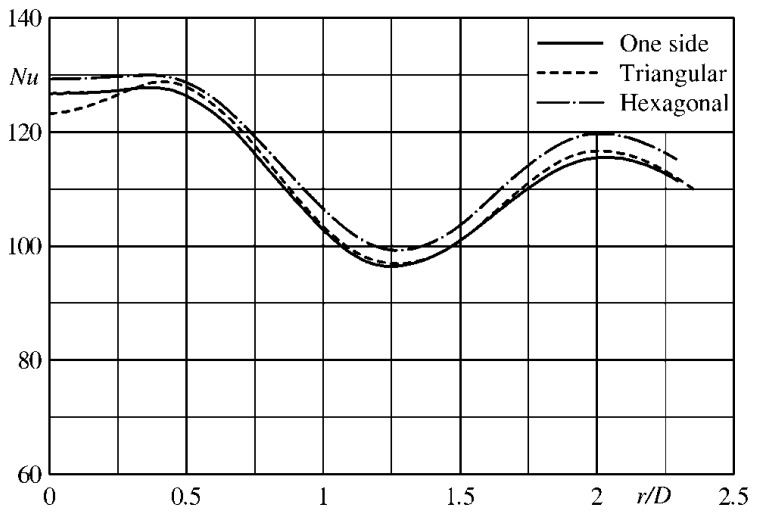
Azimuthally averaged profiles of the Nusselt number radial distributions of Figure 19 for the three heating configurations; *Re* = 30,000, *z/D* = 2. Data from Carlomagno *et al.* [7].

In Figure 20, the azimuthal averages of the Nusselt number as a function of the dimensionless radial coordinate *r* (from the jet axis) are reported for the three heating configurations and *z*/*D* = 2. The obtained averaged profiles are in quite good agreement with each other and with that of the literature for the same *z*/*D* value and, approximately, the same Reynolds number [28].

In spite of the reduction of the spatial resolution, connected to the use of a smoothing Gaussian filter and the calculation of the numerical derivatives with a relatively large step, the Laplacian sensor shows to be able to detect all the local variations of *Nu*, providing quite reliable results.

Anyhow, averages in the region close to the jet stagnation point are affected by a higher uncertainty since the number of available samples for the azimuthal average is not sufficient to smooth the noise of the computed Nusselt number distribution, there.

This partially justifies also the not exactly horizontal tangent and the larger differences between the Nusselt number profiles, at *r*/*D* = 0, in one of the curves reported in Figure 20. Nevertheless, on the average, the differences are contained within a few per cent.

Meola *et al.* [4], by measuring the adiabatic wall temperature *T_aw_* on a plate with impinging jets, observe with IRT the instabilities developing at relatively high Mach numbers *M*. The phenomenology is strongly dependent on the impingement distance *z*; in particular, for *z/D* < 6, as *M* increases, the vortex ring, which is located in the shear layer at about 1.2*D* from the jet axis, strengthens up to its highest magnitude (*M* ≈ 0.7). Then for even larger Mach numbers, the vortex ring breaks up (Widnall instability, Widnall *et al.* [36]) with the formation of secondary flow structures.

**Figure 21.**
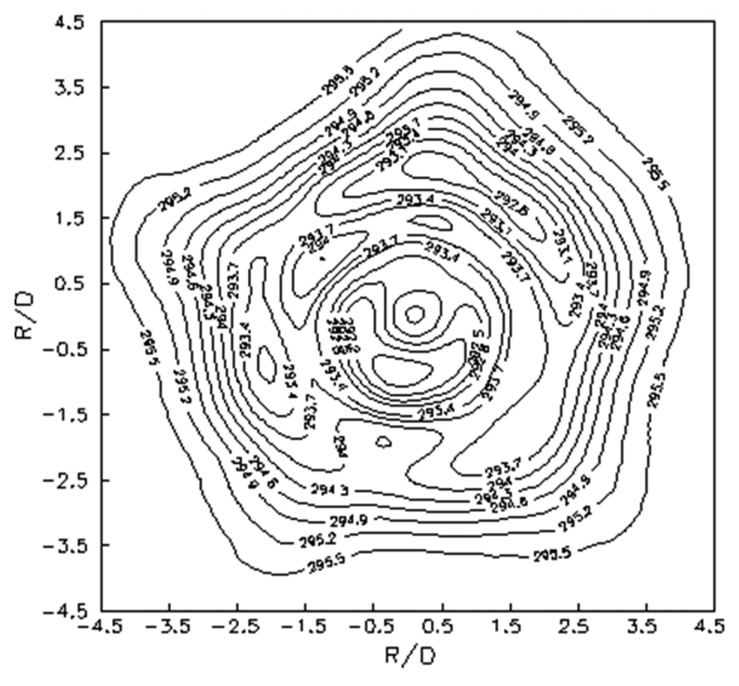
Adiabatic wall temperature on a plate with a normally impinging jet. *D* = 5 mm; *z/D* = 4; *M* = 0.78; *Re* = 86,400. From Meola *et al.* [4].

At low Mach number the *T_aw_* map is practically axisymmetric. But, as *M* increases, the vortex ring reinforces and, for *M* ≈ 0.7, breaks up when impacting onto the plate, entraining warmer ambient air and giving rise to secondary minima, with maxima in between them. This is shown in the adiabatic wall temperature map of Figure 21, which refers to *D* = 5 mm, *z/D* = 4, *M* = 0.78, *Re* = 86,400. For higher *M*, the structures first strengthen up and reach their highest magnitude and, with a further Mach number increase, they break up into numerous smaller structures, which tend to coalesce giving rise to a transient alternate circumferential movement.

Since the temperature fluctuations at *M* > 0.7 for *T_aw_* and *T_w_* are of the same order of magnitude, the azimuthally averaged radial distribution of the Nusselt number does not show additional peaks. Nevertheless, some azimuthal structures are visible in the two-dimensional map of the Nusselt number. In fact, local *Nu* contours exhibit loss of symmetry about the stagnation point, this effect being due to the transient alternate circumferential movement of the instability structures [4].

It is interesting to point out that, to perform the qualitative studies on the adiabatic wall temperature distribution such as the map presented above, the sensor to be used (on which the jet impinges) could be simply constituted by a very low thermal conductivity flat surface.

### 9.4. Rotating Channels

As it is well known, to cool gas turbine vanes and blade, air from the high pressure compressor stage goes through the hub section into the component interior and, after flowing through serpentine passages, is discharged into the main flow to provide film cooling as well. These passages are mostly made of several adjacent straight ducts, spanwise aligned along the blade and connected by 180° turns (also called *U turns*). Turns cause flow separation/reattachment and induce secondary flows so the convective heat transfer coefficient exhibits high variations with consequent wall thermal stresses. For a rotating channel, it is common usage to call *leading* the wall that goes ahead during rotation and *trailing* the one that follows.

One of the first attempts to measure convective heat transfer coefficients in a rotating air channel with IR thermography is reported by Cardone *et al.* [37]. The apparatus concept is a direct consequence of the used heated thin foil sensor. Since the foil back surface (to be viewed by the IR camera) cannot be thermally insulated, the only way to prevent high thermal losses by forced convection over this surface (see Equation (14), Section 4.1) was to have the channel rotating in a vacuum chamber. In the pioneering work by Cardone *et al.*, the spatial resolution of the results was relatively poor on account of the strong influence of the tangential conduction heat fluxes which were due to the relatively small dimension of the channel (22 mm × 22 mm). Therefore, the presented results were not the detailed ones that can be obtained with IR thermography but this work is reported because it offers a useful option to study flows into moving bodies.

A different approach, in order to reduce the relative importance of the external convection with respect to the internal one is chosen by Gallo *et al.* [17] to obtain detailed Nusselt number maps near a 180° sharp turn of a rotating U channel with the heated thin foil sensor. The authors decide to significantly increase *h* at the sensor front surface by using water as a working fluid as well as to use a much larger channel in order to reduce its rotational speed and, therefore, *q_a_* at the back surface. In this way, they are able to obtain a quite good spatial resolution and low tangential conduction in the acquired measurements.

**Figure 22.**
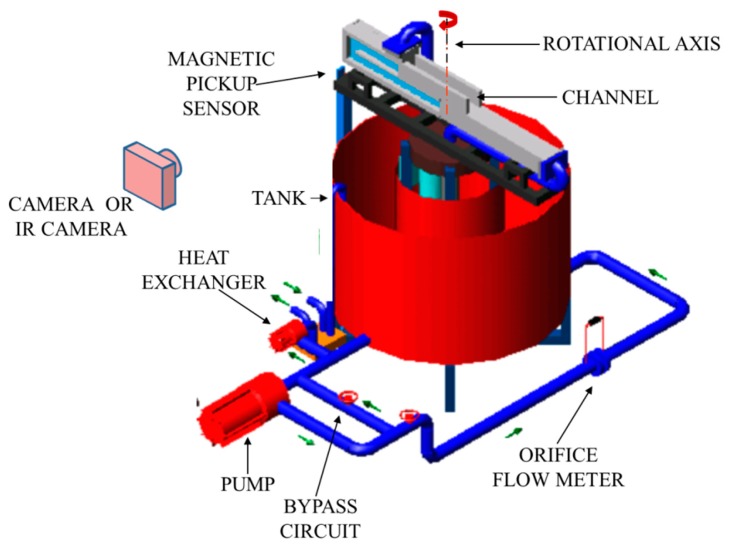
Experimental apparatus for a rotating channel of Gallo *et al.* [17].

The experimental apparatus, represented in Figure 22, consists of a Plexiglas® two-pass water channel with a sharp 180° turn, mounted on a revolving platform whose rotational speed can be continuously varied and precisely monitored in the range 0 ÷ 60 rpm. The channel has a square cross section with a side *d* = 60 mm, its length of 1200 mm ahead of the 180° turn ensuring an almost dynamically fully developed flow before the turn. The central partition wall dividing two adjacent ducts is 12 mm thick. Water from a tank is pumped through an orifice meter, a rotating hydraulic coupling and, after flowing in the test channel, is discharged back into the tank. Mass flow rate can be varied with a by-pass circuit and the inlet to channel water temperature is kept constant with a heat exchanger. A magnetic pick-up allows the synchronization of the IR image acquisition.

The apparatus is capable of simulating both Reynolds number *Re* and *Rotation number Ro* = *ω d*/*V* (where *ω* is the angular speed of the channel) values typical of turbine blades. The *d* increase and the *V* decrease (to maintain a given *Re*) allow a drastic reduction in *ω* by keeping a constant *Ro*. Results relative to the static channel (no rotation) are in good agreement with the measurements performed with air by Astarita and Cardone [10].

Heat transfer coefficients are obtained for two heating conditions (from one or both channel sides) and presented in terms of the normalised local Nusselt number *Nu*/Nu* (which can be considered also as *h*/h*). Nu* and h* are respectively the Nusselt number and the convective heat transfer coefficient values predicted by the Dittus and Bölter correlation for fully developed channel flows as interpreted by Kakac *et al.* [15].

**Figure 23.**
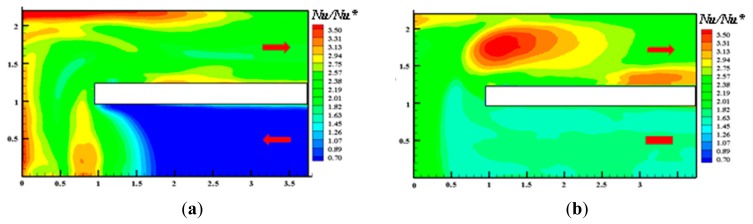
Normalised Nusselt number Nu/Nu* maps for a two-side heated rotating channel; *Re* = 20,000; *Ro* = 0.3: (**a**) Leading wall; (**b**) Trailing wall. Flow enters from bottom and exit from top. Adapted from Gallo *et al.* [17].

In Figure 23a, the normalised Nusselt number *Nu*/Nu* (as earlier defined) distribution over the leading wall, for *Re* = 20,000 and *Ro* equal to 0.3, is represented. In the inlet duct, the flow appears to be fully developed, also from the thermal point of view, since the normalised Nusselt number is practically constant. The *Nu*/Nu* values are lower than those relative to the static case and, following Gallo *et al.* [17], decrease with the increasing of the rotation number *Ro*.

In the first half of the first corner, it is possible to notice a high heat transfer zone that is caused by the inversion of the Coriolis force in the turn zone. Really in the turn zone, the radial velocity component suddenly decreases and changes sign with a consequent decrease and inversion of the Coriolis force. This inversion makes the flow separate by the trailing side, and to abruptly reattach toward the leading one, with a strong increase of the normalised Nusselt number at the reattachment point. Other low and high heat transfer zones are clearly visible in the normalised Nusselt map and their cause is explained in detail by flow field measurements made with particle image velocimetry in the work of Gallo *et al.* [38].

As it is possible to see from Figure 23b, the normalised Nusselt number distribution over the trailing wall appears to be completely different from that on the leading wall. In the inlet channel, the *Nu*/Nu* values are again uniform, but much higher than those for the static case and for the leading wall; besides, as reported by the authors [17], they tend to increase for increasing rotation number *Ro*.

In the turn region, it is possible to note that the iso-Nusselt zone tend to advance into the first corner of the turn and to insinuate in the first half of the second corner. On the second outer angle, it is possible to note a high heat transfer zone that, near the frontal wall, results as being adjacent to a relatively lower heat transfer zone. In the outlet duct, the Nusselt number distribution relative to the trailing wall exhibits two high heat transfer coefficient zones located respectively on the center and downstream near the partition wall. Again, the reason for such behavior can be found in the paper of Gallo *et al.* [38].

It should be noted that the measurements by Gallo *et al.*, are performed at relatively high Biot number because of the presence of water in the channel and since the heated tracks of the printed circuit are placed at the sensor back surface for electrical insulation reasons. Therefore, data are reduced according to the specific procedure described in Section 4.2.

### 9.5. Spray Tubes

As far as aircraft wings are concerned, de Luca and co-workers have studied both the boundary layer transition from laminar to turbulent flow on an airfoil for varying angle of attack [39] as well as the phenomena of flow separation and reattachment over a delta wings [40], both with the heated thin foil sensor.

With the same sensor, Imbriale *et al.* [41] went inside the leading edge of an airfoil to analyze wing de-icing aspects. This investigation represents a cylindrical geometry heated thin foil sensor realized with a metallic foil inserted in an *ad-hoc* fixture.

In fact, spray tubes (often called *piccolo tubes*) are amongst the most widely used anti-icing devices for wings and engine nacelles of commercial aircrafts [42]. In such devices, hot air is extracted from the compressor and blown on the inside surface of the wing leading edge, through small holes drilled in a pipe. The aim is to supply enough energy to keep the wing surface above the freezing point of water and to liquefy impinging ice crystals.

Sometimes, also to cool leading edges of turbine blades, a spray tube inside and parallel to them, with an array of aligned holes, generates a jet row which blows cold air to maintain the blade surface temperature below critical values [43]. In these devices, the impingement distance is relatively short, this problem being addressed for single jets by Carlomagno and Ianiro [35].

A piccolo tube is experimentally analyzed by Imbriale *et al.* [41]. The test article, using the fixture of Meola *et al.* [44], includes the leading edge of a NACA 0012 wing profile with a 1.50 m chord, with a spray tube located inside at 4% of the chord profile. The profile is 0.20 m span-wise long and it is stopped at about 1/10 of the chord with an open side to facilitate discharge of injected gas so to avoid recirculation effects. To allow measurements with the heated thin foil sensor, the leading edge section consists of a thin stainless steel sheet (40 μm thick) lodged inside an *ad-hoc* fixture that provides bus bars exactly shaped to the wing profile. A small indium wire is inserted between bus bars and metal sheet to assure a good electrical contact.

In particular, different spray tubes are used with a different number of holes from 3 to 5, orifice diameter *D* from 2 mm to 4 mm, and orifice pitch-to-diameter ratio *p/D* between 5 and 15. In addition, the jet inclination *φ* is varied from 0° to 50° by rotating the spray tube around its axis. The jets exit Mach number *M* is varied from 0.6 to 1.0. Herein, only results relative to 3 orifices having *D* = 4 mm, *φ* = 30°, *p/D* = 15 and M = 1 are reported.

In order to avoid measurement errors due to the surface directional emissivity decay, the camera viewing angle is always kept lower than 55°. Raw data is corrected for radiation, tangential conduction within the sensor and natural convection at the foil viewed side.

Due to the curvature of the viewed surface, to obtain the temperature distribution over the entire leading edge surface, at least two images must be acquired; temperature maps are reconstructed on the object mesh grid according to Cardone *et al.* [45]. This involves not only a different data reduction, but mainly a geometrical calibration of the IR camera. Then, before performing calculation with Equation (23), the temperature maps of *T_w_* and *T_aw_* have to be reconstructed on a 3D mesh grid from the 2D acquired IR images.

**Figure 24.**
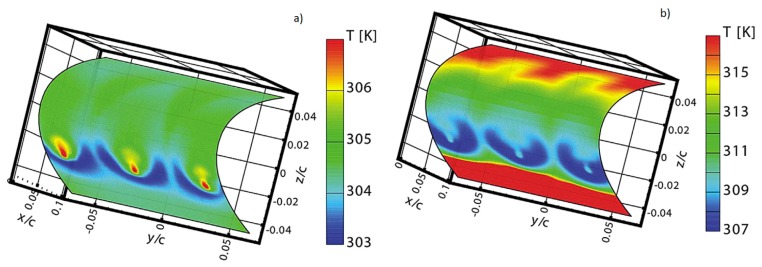
3D temperature reconstruction for *D* = 4 mm, *φ* = 30°, *M* = 1.0 and *p/D* = 15: (**a**) adiabatic wall temperature *T_aw_* (foil not heated); (**b**) wall temperature *T_w_* (heated foil), Adapted from Imbriale *et al.* [41].

An example of such a map reconstruction is given in Figure 24 for the adiabatic wall temperature *T_aw_* (Figure 24a, foil not heated) and for the wall temperature *T_w_* (Figure 24b, heated foil), taken for three aligned holes, *D* = 4 mm, inclination angle *φ* = 30°, *M* = 1.0 and orifice pitch-to-diameter ratio *p/D* = 15. Maps clearly show the jet impingement zones which are not centered with respect to chord due to the 30° jet inclination and, again, the influence of the relatively high Mach number on the adiabatic wall temperature distribution is recognized.

**Figure 25.**
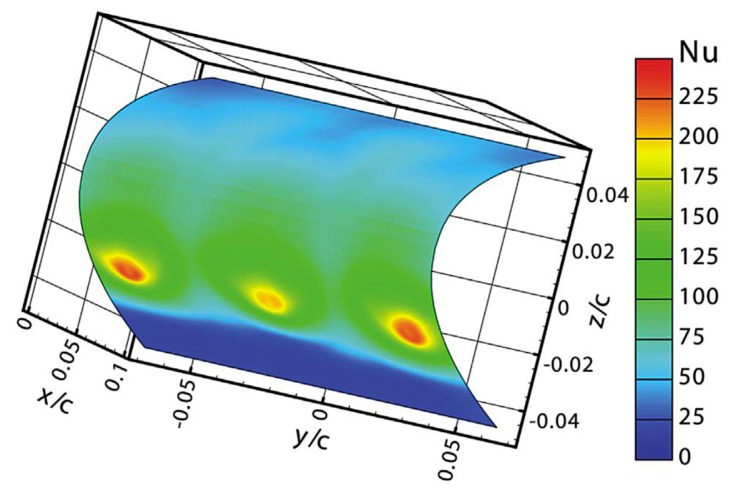
Nusselt number reconstruction for *D* = 4 mm, *φ* = 30°, *M* = 1.0 and *p/D* = 15. Adapted from Imbriale *et al.* [41].

A typical *Nu* map, for the same testing conditions of Figure 24, is shown in Figure 25. The impinging jets entail very high Nusselt number values with local peaks in a small region matching with the jets centers; so, these peaks clearly locate the area of jet impingement on the front-side surface. Even though the holes are perfectly circular, the high *Nu* region somehow stretches in chord-wise direction. This behavior is due to the jets inclination with respect to the foil surface; in fact, only jets with inclination *φ* either equal to 0° or 65° are perpendicular to the foil; for other *φ* values, the impingement is affected by inclination effects.

In addition, variations in the *Nu* distribution are present on the backside region (*z/c* > 0.2). In particular, it is possible to recognize local *Nu* increase in the span-wise direction between two contiguous jets, at the same locations where the two green streaks are visible in the wall temperature maps of Figure 24. This behavior resembles the fountain effect, already described in literature [46]. However, some fundamental differences regarding position and appearance of such local maxima exist. In fact, the fountain effect, described in literature, is exactly localized between contiguous jets; while, in the present case, local heat transfer maxima seem to originate between jets but they extend and strengthen on the backside region, far from the impingement.

### 9.6. Chevron Nozzles

The great implications that vortical features can have on surface heat transfer rate and distribution motivated the flourishing of countless investigations devoted to passive and active strategies to enhance the heat flux between a jet and an impinged surface.

Passive strategies are mainly based on the shape of the nozzle. In particular, Gao *et al.* [47] show that, for a nozzle-to-plate distance of 4 diameters, triangular tabs placed around a circular orifice (*chevron nozzle*) lead to an enhancement higher than 25% with respect to the round configuration for the same nozzle-to-plate distance.

Besides in recent years, a special attention is devoted to a particular, but very relevant, technique in the field of modern experimental thermo-fluid-dynamics, namely Tomographic Particle Image Velocimetry (Tomo-PIV). The outcomes of its visualization and flow inspection capabilities can be compared with those of recent advanced computational methods. In particular, while infrared thermography gives the outcome of the flow field on the skimmed over surface, Tomo-PIV contributes to understand the reasons for such an outcome.

Tomographic PIV is based on the double-pulsed laser illumination of a fluid volume seeded with particles; the particles distribution is reconstructed from multiple camera views and then the volumetric flow field is obtained through volume cross-correlation. Tomographic PIV is proved to be capable of high resolution volumetric flow measurements which allow for the computation also of derivative quantities giving a detailed description of vortex and turbulence dynamics.

**Figure 26.**
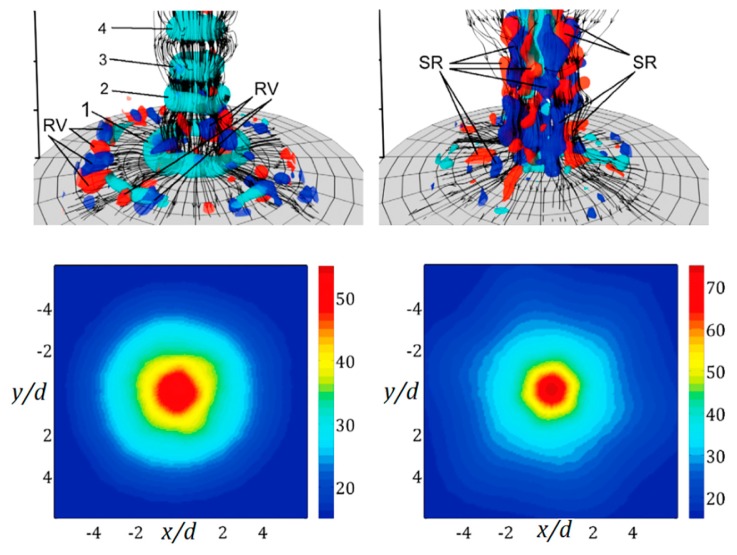
Instantaneous flow patterns (**top**) and Nusselt number distributions on the impinging plate (**bottom**) for a cylindrical (**left**) and a chevron (**right**) nozzle; *Re* = 5000, *z/D* = 4; RV radial vortices, SR streamwise structures. Adapted from Violato *et al.* [48].

The chevron arrangement is also exploited in [48] where it is shown that, compared to the circular nozzles, heat transfer at impingement is enhanced by using the chevron ones; in the center of the impinged area, the chevron jet exhibits heat transfer values up to 44% higher than those provided by the circular jet. This is shown by the bottom images of Figure 26 where the maps of the average Nusselt number on the impinging plate for *Re* = 5000 and *z/D* = 4 (symbols as in Section 9.3) are presented. This event is addressed to the development in the free jet region of streamwise vortices which, compared with the toroidal vortices of the circular nozzles, are associated with a deeper penetration of turbulent induced mixing and to a higher arrival speed (see top images of Figure 26).

The coupling of the inspection capabilities of the two experimental techniques, such as IR thermography and Tomographic PIV, allows for a deeper understanding of the involved thermo-fluid-dynamic phenomena in this very complex flow field.

## 10. Conclusions

This paper has the aim of reviewing and assessing the most common heat flux sensors, which can be used with infrared thermography to measure convective heat transfer coefficient distributions between a body and a fluid flowing over it. After recalling the basic principles that make IR thermography work, the various heat flux sensors to be used with it are presented and discussed, namely: heated thin foil, gradient sensor, Laplacian sensor, thin film and thin skin. For each sensor, the basic model, its limits, the data analysis and the experimental procedure are delineated.

Applications to complex thermo-fluid-dynamic flows that range from natural convection to hypersonic flows are finally outlined by describing some of the heat flux sensors exploitations performed by the authors' research group at the University of Naples Federico II.

For all the presented applications, the infrared adopted technique proves its capability to accurately measure the convective heat transfer coefficient distributions generated by the fluid flows in the examined complex geometries and to be a very effective investigation tool for thermo-fluid-dynamic experimental research.

When compared to standard techniques, the use of an infrared camera as a temperature transducer in convective heat transfer measurements appears advantageous from several points of view. In fact, since the IR camera is two-dimensional, with current systems having up to about 1 M pixels per frame, besides producing a whole temperature map, IRT allows an easier evaluation of errors due to radiation and tangential conduction. The camera does not disturb the phenomena being measured and does not alter conduction through test article embedded sensors and wiring, it has high sensitivity (down to 10 mK) and low response time (down to 20 ms). As such, IR thermography can be effectively exploited to measure convective heat fluxes (with either steady, or transient, techniques) even in circumstances where they undergo drastic variations.

## Figures and Tables

**Table 1.** Quantities to be measured in the various heat flux sensors.

**Sensor**	**Measured Quantity**	
Heated thin foil	*T_w_*	*T_1_*
Gradient sensor	*T_w_* – *T_1_*	*N. A.*
Laplacian sensor	∇^2^*T_w_*	∇^2^*T*_1_
Thin film	*T_w_*(*t*)	*N. A.*
Wall calorimeter	*T_w_*(*t*)	*T_1_*(*t*)

## List of Symbols

**Roman letters**
*A*: Temperature amplitude Equation (39)
*A_q_*: Heat flux amplitude, Equation (38)
*c*: Sensor specific heat, Airfoil chord
*c_p_*: Fluid specific heat at constant pressure
*C_1_*: First radiation constant
*C_2_*: Second radiation constant
*d*: Channel diameter
*D*: Nozzle diameter
*E_b_*: Emissive power of black body
*^f^*: Attenuation factor Equation (39)
*^F^*: Temperature modulation transfer function
*g*: Gravity acceleration
*G*: Heat generation per unit volume
*I*: Spectral emissive power
*h*: Convective heat transfer coefficient
*k*: Thermal conductivity coefficient
*p*: Pitch, Dimensionless quantity, Equation (29)
*q*: Heat flux
*r*: Radial coordinate
**ℛ**: Dimensionless wavelength
*s*: Sensor thickness, Thickness
*t*: Time
*t_m_*: Maximum measurement time (thin film)
*T*: Temperature
*V*: Fluid velocity
*w*: Width
*x*: Coordinate
*y*: Coordinate
*z*: Coordinate Nozzle-to-plate-distance

**Greek letters**
α: Sensor thermal diffusivity coefficient, Angle of attack
α*_f_*: Fluid thermal diffusivity coefficient
α*_r_*: Absorptivity coefficient
*β*: $\beta =h\sqrt{t/\left(\rho_{s}ck_{s}\right)}$
*ε*: Emissivity coefficient
*λ*: Wavelength of electromagnetic wave
*ν*: Fluid kinematic viscosity coefficient
ξ: $\xi =x/2\sqrt{\alpha t}$
Ξ: Dimensionless quantity, Equation (31)
Π: Dimensionless ratio, Equation (45)
*^θ^*: Dimensionless temperature, Equation (26)
Θ: Dimensionless quantity, Equation (44)
*ρ*: Mass density
*ρ_r_*: Reflectivity coefficient
*σ*: Stefan-Boltzmann constant
*τ_r_*: Transmissivity coefficient
*φ*: Temperature difference, *φ* (*t*) = *T_w_*(*t*) − *T_wi_*
*φ*: Jet inclination to airfoil chord
*ω*: Angular speed

**Subscripts**
*a*: Ambient
*aw*: Adiabatic wall
*b*: Black body
*c*: Convective, Copper
*f*: Fluid, Fiberglass
*j*: Joule
*m*: Mean
*r*: Radiative, Reference
*s*: Sensor
*t*: Tangential, Total
*x*: Local
*w*: Wall
*ϖ_x_*: Wave number

**Apices**
*m*: At maximum emissive power
*: As per Dittus and Bölter correlation

**Dimensionless groups**
*Bi*: Biot number, *hs/k_s_*
*Bi_x_*: Local Biot number, *hx/k_s_*
*Fo*: Fourier number, *^αt^/^s^*^2^
*Fo_x_*: Local Fourier number, *αt/x^2^*
*Fo_ϖ_*: Modified Fourier number, $\varpi_{x}^{2}\alpha t$
*M*: Mach number
*Nu*: Nusselt number, *hd/k_f_*
*Pr*: Prandtl number, *c_p_μ/k_f_*
*Ra*: Rayleigh number, *gβqx^4^*/(*ν α_f_k_f_*)
*Re*: Reynolds number, *Vd/ ν*
*Ro*: Rotational number, *ν d/V*
*St*: Stanton number, *h/*(*ρ_f_c_p_V*)

## References

[1] Astarita T., Carlomagno G.M. (2013). Infrared Thermography for Thermo-Fluid-Dynamics.

[2] Schlichting H. (1979). Boundary-Layer Theory.

[3] Shapiro A.H. (1954). The Dynamics and Thermodynamics of Compressible Fluid Flow.

[4] Meola C., de Luca L., Carlomagno G.M. (1996). Influence of shear layer dynamics on impingement heat transfer. Exp. Therm. Fluid Sci..

[5] Rohsenow W.M., Hartnett J.P. (1973). Handbook of Heat Transfer.

[6] Carlomagno G.M., de Luca L., Yang W.J. (1989). Infrared Thermography in Heat Transfer. Handbook of Flow Visualization.

[7] Carlomagno G.M., Discetti S., Astarita T. (2011). Experimental assessment of a new heat flux sensor for measuring convective heat transfer coefficients. Quant. Infr. Therm. J..

[8] Roger M. (2007). A periodic-transient method for high-resolution heat transfer measurement on two dimensional curved surfaces. J. Heat Transf. Trans. ASME.

[9] Cardone G., Carlomagno G.M. Convezione naturale su lastra piana nei regimi stazionario ed instazionario.

[10] Astarita T., Cardone G., Carlomagno G.M. (2002). Convective heat transfer in ribbed channels with a 180 degrees turn. Exp. Fluids.

[11] Carlomagno G.M., Cardone G. (2010). Infrared thermography for convective heat transfer measurements. Exp. Fluids.

[12] Carlomagno G.M., Nese F.G., Cardone G., Astarita T. (2004). Thermo-fluid-dynamics of a complex fluid flow. Infrared Phys. Technol..

[13] Perry J.H. (1963). Chemical Engineers' Handbook.

[14] Kays W.M., Crawford M.E. (1993). Convective Heat and Mass Transfer.

[15] Kakac S., Shah R.K., Aung W. (1987). Handbook of Single Phase Flow Convective Heat Transfer.

[16] Astarita T., Cardone G. (2000). Thermofluidynamic analysis of the flow in a sharp 180 degrees turn channel. Exp. Therm. Fluid Sci..

[17] Gallo M., Astarita T., Carlomagno G.M. (2007). Heat transfer measurements in a rotating two-pass square channel. Quant. Infr. Therm. J..

[18] Kaiser R., Du Puits R. (2014). Local wall heat flux in confined thermal convection. Int. J. Heat Mass Transf..

[19] Douglas B. (1972). Selecting Unsteady Heat Flux Sensors. Instr. Contr. Syst..

[20] Rainieri S., Bozzoli F., Pagliarini G. (2004). Wiener filtering technique applied to thermographic data reduction intended for the estimation of plate fins performance. Exp. Therm. Fluid Sci..

[21] Carslaw H.S., Jaeger J.C. (1959). Conduction of Heat in Solids.

[22] Gulhan A., Schutte G., Stahl B. (2008). Experimental Study on Aerothermal Heating Caused by Jet-Hypersonic Crossflow Interaction. J. Spacecr. Rocket..

[23] Cook W.J., Felderman E.J. (1966). Reduction of data from thin-film heat-transfer gages: A concise numerical technique. AIAA J..

[24] De Luca L., Cardone G., de la Chevalerie D.A., Fonteneau A. (1995). Viscous interaction phenomena in hypersonic wedge flow. AIAA J..

[25] Mulcahy J.M., Browne D.J., Stanton K.T., Diaz F.R.C., Cassady L.D., Berisford D.F., Bengtson R.D. (2009). Heat flux estimation of a plasma rocket helicon source by solution of the inverse heat conduction problem. Int. J. Heat Mass Transf..

[26] De Felice G., de Luca L., Carlomagno G.M. La misura dei flussi convettivi nel caso di distribuzioni non uniformi.

[27] Jepps G. (1965). Heat Conduction in Single-layer and Double-layer Walls, with Boundary Conditions. Australian Aeronautical Research Committee Report ACA-66.

[28] Carlomagno G.M., de Luca L. Infrared thermography for flow visualization and heat transfer measurements.

[29] Sparrow E.M., Gregg J.I. (1956). Laminar free convection from a vertical plate with uniform surface heat flux. Trans. ASME.

[30] De Luca L., Cardone G., Carlomagno G.M. Image Restoration in Thermo-Fluid-Dynamic Applications of IR Digital Imagery.

[31] De la Chevalerie D.A., Fonteneau A., de Luca L., Cardone G. (1997). Görtler-type vortices in hypersonic flows: The ramp problem. Exp. Therm. Fluid Sci..

[32] De Luca L., Cardone G., Carlomagno G.M., de la Chevalerie D.A., de Roquefort T.A. (1992). Flow visualization and heat transfer measurement in a hypersonic wind tunnel. Exp. Heat Transf..

[33] Meola C., de Luca L., Carlomagno G.M. (1995). Azimuthal instability in an impinging jet-adiabatic wall temperature distribution. Exp. Fluids.

[34] Carlomagno G.M., de Luca L., Veret C. (1987). Heat Transfer Measurements by Means of Infrared Thermography. Flow Visualization IV.

[35] Carlomagno G.M., Ianiro A. (2014). Thermo-fluid-dynamics of submerged jets impinging at short nozzle-to-plate distance: A review. Exp. Thermal Fluid Sci..

[36] Widnall S.E., Bliss D.B., Tsai C. (1974). The instability of short waves on a vortex ring. J. Fluid Mech..

[37] Cardone G., Astarita T., Carlomagno G.M. (1998). Wall heat transfer in static and rotating 180 degrees turn channels by quantitative infrared thermography. Rev. Gen. Therm..

[38] Gallo M., Astarita T., Carlomagno G.M. (2012). Thermo-fluid-dynamic analysis of the flow in a rotating channel with a sharp “U” turn. Exp. Fluids.

[39] De Luca L., Carlomagno G.M., Buresti G. (1990). Boundary-layer diagnostics by means of an infrared scanning radiometer. Exp. Fluids.

[40] De Luca L., Guglieri G., Cardone G., Carlomagno G.M. (1995). Experimental analysis of surface flow on a delta wing by infrared thermography. AIAA J..

[41] Imbriale M., Ianiro A., Meola C., Cardone G. (2014). Convective heat transfer by a row of jets impinging on a concave surface. Int. J. Thermal Sci..

[42] Cebeci T., Kafyeke F. (2003). Aircraft Icing. Ann. Rev. Fluid Mech..

[43] Iacovides H., Launder B.E. (2007). Internal blade cooling: The Cinderella of computational ad experimental fluid dynamics research in gas turbines. Proc. Inst. Mech. Eng. Part A J. Power Energy.

[44] Meola C., Carlomagno G.M., Riegel E., Salvato F. An Experimental Study of an Anti-Icing Hot Air Spray-Tube System.

[45] Cardone G., Ianiro A., dello Ioio G., Passaro A. (2012). Temperature maps measurements on 3D surfaces with infrared thermography. Exp. Fluids.

[46] Iacovides H., Kounadis D., Launder B.E., Li J., Xu Z. (2005). Experimental study of the flow and thermal development of a row of cooling jets impinging on a rotating concave surface. J. Turbomach..

[47] Gao N., Sun H., Ewing D. (2003). Heat transfer to impinging round jets with triangular tabs. Int. J. Heat Mass Transf..

[48] Violato D., Ianiro A., Cardone G., Scarano F. (2012). Three-dimensional vortex dynamics and convective heat transfer in circular and chevron impinging jets. Int. J. Heat Fluid Fl..

